# Nonconceptus Mechanisms of Prenatal Alcohol Exposure That Disrupt Embryo-Fetal Development: An Integrative View

**DOI:** 10.35946/arcr.v45.1.07

**Published:** 2025-07-16

**Authors:** Susan M. Smith

**Affiliations:** UNC Nutrition Research Institute and Department of Nutrition, University of North Carolina at Chapel Hill, Kannapolis, North Carolina

**Keywords:** alcohol, prenatal alcohol exposure, fetal alcohol spectrum disorder, inflammation, microbiome, epigenetics, placenta, paternal alcohol exposure, metabolism, anemia

## Abstract

**PURPOSE:**

Prenatal alcohol exposure (PAE) is a leading cause of persistent neurodevelopmental disability, with additional adverse consequences to the offspring’s growth, metabolism, cardiovascular health, and immunity, among others. Alcohol disrupts offspring development through myriad mechanisms, many of which involve direct interactions between alcohol and the embryo and fetus (i.e., the conceptus). This limited narrative review instead focuses on mechanisms that are exogenous to the fetus. Many of these are relatively unexplored and are also mechanistically interrelated. Thus, they represent novel opportunities for the design of interventions that ameliorate alcohol-related pathologies.

**SEARCH METHODS:**

Literature from 2020 to October 2024 was searched using the terms “fetal alcohol spectrum disorder”[MeSH] OR “fetal alcohol”[Ti/Ab] with the filter “review.” These reviews were inspected to extract nonfetal mechanisms of alcohol. Literature from 2000 to October 2024 was then searched in PubMed, Embase, and Google Scholar for seven mechanisms, using the search terms “fetal alcohol spectrum disorder OR fetal alcohol” AND one of the following: “placenta,” “paternal,” “metabolism OR insulin OR amino acid,” “inflammation OR neuroinflammation OR cytokine,” “epigenetic,” “iron OR iron deficiency OR anemia,” “microbiome.” Only primary research articles, both clinical and preclinical, were included.

**SEARCH RESULTS:**

The literature scan identified seven mechanisms for which targeted literature searches were conducted. These searches yielded relevant studies that explored mechanisms involving the microbiome (*n* = 5 studies), inflammation (*n* = 72 studies), epigenetics (*n* = 30 studies), paternal alcohol exposure (*n* = 34 studies), placenta (*n* = 53 studies), metabolism (*n* = 37 studies), and functional iron deficiency (*n* = 23 studies).

**DISCUSSION AND CONCLUSIONS:**

Exogenous mechanisms of alcohol’s teratogenicity are intertwined. Alcohol remodels the maternal enteric microbiome, with potential consequences to fetal immune function, nutrient availability, and brain development. Microbial endotoxins may further magnify alcohol’s proinflammatory actions. This inflammation might also drive a fetal anemia associated with PAE. Alcohol alters maternal and fetal metabolism and could limit fetal nutrient availability. This altered metabolism could also reprogram placental and fetal epigenetics, as could paternal exposure to alcohol. Both epigenetic effects and inflammation can impair placental function and modulate the placenta–brain axis that modulates brain development. The review discusses limitations in the current understanding of these mechanisms and highlights future research avenues that would provide clarity and inform future interventions.

## Introduction

Prenatal alcohol exposure (PAE) is the leading known cause of preventable neurodevelopmental disability. The clinical manifestations of PAE, fetal alcohol spectrum disorders (FASD), are primarily characterized by cognitive and behavioral deficits, but also feature craniofacial anomalies; growth deficits; and metabolic, endocrine, immune, musculoskeletal, and cardiovascular disorders that persist through the life span. 1,2 In the United States, 1% to 5% of first graders meet the diagnostic criteria for FASD. 3 This aligns with the rates of gestational alcohol exposure, with 14% of pregnant women self-reporting drinking and 5% reporting binge drinking (defined as four or more drinks on one occasion) in the prior 30 days. 4 These rates have not declined despite widespread efforts at prevention. 4 Thus, there is high interest in gestational and postnatal interventions that attenuate or even prevent alcohol’s damage.

The design and application of interventions are informed by the identification of the underlying mechanisms by which alcohol (i.e., ethanol) induces its pathologies. Alcohol’s pathology originates from two distinct metabolic and pharmacological mechanisms. In the first, alcohol oxidation disrupts metabolic processes within organs that catabolize alcohol, including the liver, enterocytes, astrocytes, and other cell lineages. 5 These disruptions further affect other organs via secondary metabolites (e.g., acetaldehyde, acetate, ketones) 6 and spill-over physiological consequences, such as altered lipid metabolism. 7,8 Alcohol’s pharmacological mechanisms are also well understood. Although alcohol was originally posited to have membrane dissolution properties, 9 molecular-level studies revealed saturable effects representing specific alcohol–protein interactions. Although there is no single alcohol receptor, protein structural studies revealed that alcohol physically interacts with hydrophilic regions or “pockets” within select proteins. 10,11 These interactions induce conformational changes within the protein that alter its activity. How any given protein responds to alcohol binding must be defined experimentally. For some proteins, alcohol binding increases their activity by prolonging interactions with ligands or partnering proteins. For other proteins, alcohol reduces their activity by blunting those interactions. 10,11 These binding pockets can be mapped using longer-chain alcohols (i.e., the Richardson effect 9 ), and for each protein there is a size cut-off beyond which larger alcohols cannot bind and modulate protein activity. Known target proteins that have developmental relevance include receptors for gamma-aminobutyric acid, glycine, serotonin, and *N*-methyl-d-aspartate (NMDA); the inwardly rectifying potassium channel Kir2.1; the L1 cell adhesion molecule; and G-protein signaling. 10–14 Nearly every tissue and pathway responds to alcohol; however, to date too few of these alcohol-binding proteins have been identified for further investigation. Application of the protein structure modeling program Alpha-Fold to this question may facilitate the discovery of novel alcohol targets. 15

A significant challenge for mechanistic studies of PAE is that this plethora of targets produces seemingly contradictory effects that are a function of dose and duration of exposure, gestational stage, cell lineage and differentiation status, and even species. This complicates identification of alterations that represent the primary mechanisms of action and the most impactful targets for intervention. Expanding this complexity are more recent demonstrations that PAE interacts not only with the conceptus, but also with the mother, biological father, placenta, and maternal microbiotas. 7,16–18 Moreover, its effects are preconceptual as well as gestational. 17,18 An additional challenge is that not all responses to alcohol are adverse. Some are compensatory and others protective. For example, a protein’s abundance may increase to compensate for alcohol’s suppression of its activity, as is the case with the NMDA receptor. 19 Additionally, the PAE-induced activation of intracellular protein degradation (i.e., autophagy) protects against metabolic stress by providing extra nutrients. 20 Thus, a mechanism’s relevance is ultimately defined through the testing of a targeted and specific intervention that prevents the broadest range of alcohol’s downstream consequences, especially in response to a single acute exposure. A caveat here is that some interventions do not directly normalize an underlying pathology but instead induce compensatory processes that facilitate recovery. Because most mechanistic studies are performed in nonhuman models, demonstrations that a mechanism is conserved across multiple species and taxa endorses its relevance for human development.

**Figure 1:**
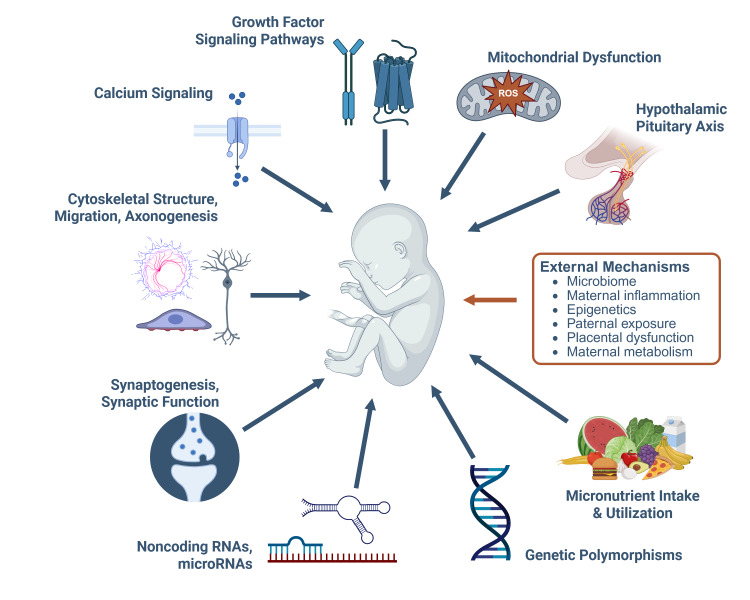
Summary of key mechanisms that underlie alcohol’s teratogenicity, both intrinsic and extrinsic to the conceptus. Mechanisms that are external to the conceptus are presented in the orange box and are the focus of this review. Figure generated in BioRender. *Note:* ROS, reactive oxygen species.

Despite these challenges, an extensive literature documents numerous mechanisms by which PAE directly interacts with the conceptus to alter its development. Many of these involve alcohol’s pharmacological actions upon cellular processes (see Figure 1).

- Dysregulation of growth factor signals—such as target of rapamycin complex 1 (TORC1), insulin-like growth factor, and hedgehog—suppresses anabolic processes, including proliferation and ribosome biogenesis and initiate apoptosis.^21^–^25^
- Suppression of mitochondrial oxidative processes reduces energy generation and, when extended, increases the production of free radicals that initiate oxidative stress.^26^–^28^
- Alterations occur in the neuroendocrine and hypothalamicpituitary-adrenal axis.^29^,^30^
- Reduced intake and/or utilization of micronutrients, including choline, folate, iron, retinoids, and zinc, causes maternal and fetal deficiencies.^31^–^34^
- Genetic polymorphisms modulate alcohol metabolism and fetal vulnerability to alcohol’s teratogenicity.^35^,^36^
- Generation of microRNAs and other noncoding RNAs that circulate in the mother and fetus modulates cellular activity.^37^,^38^
- Disruptions occur in the neurotransmitter systems required for synaptic formation, reinforcement, and plasticity, as well as for subpopulation expansion and organization within the brain.^39^–^42^
- Alterations in cytoskeletal and cell adhesion interactions disrupt cell migration, axonal pathfinding, and synaptogenesis.^43^,^44^
- Activation of intracellular calcium transients activates or suppresses downstream signaling processes, as shown for neural crest and trophoblasts.^14^,^45^

A discussion of all these mechanisms is beyond the scope of this review. Instead, the review focuses on a neglected aspect of alcohol’s actions—namely, its impact on those processes that are external to the conceptus and yet make important contributions to its development. These external forces include the microbiome, inflammation, epigenetics, paternal alcohol exposure, placenta, maternal metabolism, and functional iron deficiency. For several of these (i.e., microbiome, metabolism) their contributions to the nonpregnancy state are well understood; however, this knowledge has yet to be widely applied to PAE. The review also highlights limitations in current understanding of those mechanisms and areas for future research opportunities. It also explores how these mechanisms are interwoven and thus represent new avenues for potential interventions. The goal is to bring renewed attention to these mechanisms and expand understanding of alcohol’s teratogenicity.

## Search Methods and Results

Literature from 2020 to October 2024 was searched using the terms “fetal alcohol spectrum disorder[MeSH] OR fetal alcohol[Ti/Ab]” with the filter “review.” These review articles were then inspected to extract nonfetal mechanisms of alcohol’s teratogenicity. Using this extracted list, three databases—PubMed, Embase, and Google Scholar—were searched to identify relevant primary literature. Search dates spanned the prior quarter-century (2000 to October 2024) and were performed in October 2024; initial searches retrieving fewer than 40 articles were expanded to the database’s beginning (1981). Separate searches were conducted for each mechanism. Search terms were “fetal alcohol spectrum disorder[MeSH] OR fetal alcohol[Ti/Ab]” AND one of the following: “microbiome,” “inflammation OR neuroinflammation OR cytokine,” “epigenetic,” “paternal,” “placenta,” “metabolism OR insulin OR amino acid,” and “iron OR iron deficiency OR anemia.” Search results did not differ if “prenatal” was substituted for “fetal.” Both clinical and preclinical studies were included, as were primary research articles and reviews. The reference lists from those articles were then reviewed to retrieve additional papers not identified in the search. From all of these articles, only primary research articles are discussed in this review; articles were excluded that were reviews, were not in English or did not address PAE (see Table 1 ).

## Results of the Reviewed Studies

### Microbiome and Inflammation

#### Microbiome contributions to the effects of PAE

The microbiota comprises the bacteria, archaea, prokaryotes, viruses, and phages present on every internal and external body surface. Advances in bulk DNA sequencing have enabled the identification of these micro-organisms and their significant roles in the host organism’s health. 46 The healthy gut lumen is anaerobic and predominantly populated by microbes requiring such an environment (i.e., obligate anaerobes). Heavy alcohol consumption (defined as five or more drinks per day or 15 or more drinks per week for men and four or more drinks per day or eight or more drinks per week for women 47 ) shifts this dynamic to enrich for facultative anaerobes that also thrive under aerobic conditions. 48 This included increases in Proteobacteria and reductions in *Ruminococcus* and *Prevotella*. 48 These population shifts reflect changes in the microbiota’s metabolic profile, most notably reducing the production of short-chain fatty acids (SCFAs), such as acetate and butyrate, that support the enteric immune system, strengthen the intestinal barrier, and suppress the growth of pathogens, such as *Clostridioides difficile*. 49 Alcohol exposure, even from a single binge event, also enhances gut permeability, which enables endotoxins that are derived from the resident gram-negative bacteria (i.e., *Salmonella*, *Escherichia coli*) to enter the bloodstream. 50 These endotoxins and fragments of microbial DNA drive a persistent, systemic inflammation that further contributes to alcohol’s pathogenicity. 51

The microbiome also makes important contributions during pregnancy. During pregnancy, the mother’s gut microbiota is remodeled to enhance nutrient availability and immune tolerance and thus support fetal development. 52 Maladaptive changes to the mother’s microbiota characterize pathological conditions, including gestational diabetes, preeclampsia, fetal growth restriction, and preterm birth. 52 Communication between the gut microbiome and the developing fetal brain may affect the offspring’s behavior, 53 and microbiota-derived biosignatures have been reported for neurodevelopmental disorders, such as autism and attention deficit disorder. 54,55 The maternal microbiota also shapes the maturation of the offspring’s innate (nonspecific) and adaptive (pathogen-specific) immune systems, whereas its maladaptation contributes to immune-related diseases. 56,57 How the maternal microbiota influences the offspring is incompletely understood, but includes indirect effects through microbiota interactions with the maternal enteric immune system 53–55 and direct effects through its metabolites that enter the maternal–fetal circulation. 49,53,54 Additionally, the enteric, vaginal, breast, and oral microbiota all seed the infant’s microbiota and could be modified by alcohol exposure. 52,56,57

A few limited studies have investigated the impact of PAE on the maternal and offspring enteric microbiota; alcohol’s effects on microbiota outside of the gut (e.g., vaginal, breast, oral) remain unexplored. In preclinical studies, alcohol reduced the overall community diversity (beta-diversity) of the mother’s fecal microbiome, seen in reduced abundance of *Lactobacilli* and the butyrate producers Ruminococcaceae and Lachnospiraceae. 58 Similar population shifts were described for the nonpregnancy state following heavy alcohol consumption 48 and were associated with metabolic shifts and SCFA losses that could worsen the gut barrier and immune function. 48 The effects of PAE extended to these rat mothers’ offspring, whose fecal microbiota at weaning also had an altered community structure compared with nonexposed offspring. 58 However, these changes were quite distinct from their mothers’, possibly reflecting differences in diet, gut maturity state, and alcohol exposure. Thus, these PAE offspring had reduced abundance of the SCFA producer Bifidobacteriaceae, 58 reductions in fecal butyrate (but not acetate or propionate), 59 and expansions of genera within Bacteriodales, notably *Alistipes* and *Parabacteroides*; 58 these changes have been linked elsewhere to inflammation and depressive behaviors. 53–57 These changes persisted into adulthood, indicating that PAE’s effects on the offspring’s enteric microbiome community structure were long lasting. 58–60 PAE also reduced fecal community diversity when restricted to two binge exposures at late term. 61 Adolescent offspring again had reduced *Bifidobacterium* and accompanying enrichments in other SCFA producers (*Lactobacillus*, *Blautia, Muribaculaceae*) that might represent a compensatory response. The abundance of these latter two populations was positively correlated with performance in the rotarod and elevated plus maze tests that assess motor coordination and anxiety-related behavior, respectively. Although these findings were obtained in rodents, gut physiology is largely conserved in rodents and humans. 62 Moreover, these microbial populations have conserved functions across mammalian species and thus may exert similar effects in humans. 62

An alternate approach to interrogate the impact of alcohol-microbiota interactions is to focus on the metabolites they produce. The microbiota has metabolic actions that affect maternal and fetal nutrient needs and the abundance of circulating metabolites. 52–57 Untargeted analyses of the metabolome in a mouse model of PAE identified an alcohol-associated microbiota biosignature in late-term maternal plasma that was enriched in certain organic compounds (i.e., plant phenolics, plant steroids, and indoles) and reduced in eight secondary bile acids. 63 Many of these compounds crossed the placenta to circulate in the fetal brain and liver. Plant phenolics and some indoles may have protective actions as free radical scavengers and xenobiotic response modulators. However, other metabolites, including oxindole, indolepropionate, and 4-ethylphenylsulfate, have been implicated in neurological disorders, such as anxiety, depression, and autism. 55,64 Several of these protective phenolics and indoles were reduced in maternal blood taken from alcohol-exposed pregnancies, and their abundance positively correlated with infant length and head circumference. 65

The microbiota’s impact can also be estimated using a computational approach that determines the population’s composition based on 16S ribosomal RNA (rRNA) sequencing, to extrapolate the microbiota genomes and the putative biochemical pathways that might be present. This approach was used to analyze the fecal microbiome of alcohol-exposed rat mothers and suggested potential differences in the organic acid and pentose phosphate pathways used to generate SCFAs. 60 For their offspring, the analyses predicted possible differences in cofactor biosynthesis (e.g., thiamine, cobalamin, *myo*-inositol) and carbohydrate metabolism (hemicellulose, hexose sugars). A similar extrapolation from a short late-term binge exposure highlighted shifts in the pathways involving fatty acid and bile acid metabolism within the fecal microbial populations of the offspring. 61 Reductions in plasma secondary bile acids, which are made by the microbiota, contributed to the metabolite biosignature of alcohol-exposed pregnant mice. 63

Although it is tempting to speculate that such microbiota changes might contribute to, for example, the chronic inflammation, growth deficits, and behavioral and metabolic alterations associated with PAE, such conclusions are currently based on associations rather than demonstrations of causation. An emphasis on direct analyses, such as sampling the colon and cecum instead of feces, and sequencing the whole genome instead of 16S rRNA, would provide direct information on the functional relevance of these microbiota changes. 66 Metabolomic analysis of the colonic and cecal contents would lend functional support, as would direct testing of lead metabolite candidates. Conclusive studies on microbiota contributions will require germ-free and fixed-microbiota (gnotobiotic) animals, including the transplant of alcohol-adapted microbiomes into both pregnant dams and their offspring. However, such work has its own challenges. Additional caveats include the influences of litter, cohousing, and diet composition. 66,67 Nonetheless, carefully designed studies will inform the microbiota contributions to the effects of PAE as they have done for alcoholic liver disease. 51

#### Inflammatory contributions to the effects of PAE

Mechanistically related to alcohol’s impact on the microbiota is alcohol’s consistent association with chronic systemic and neuronal inflammation in both mother and fetus. 68,69 For example, monocytes from cord blood of alcohol-exposed pregnancies are hyperresponsive to agonists of the toll-like receptors (TLR2, TLR4) that initiate immune responses to circulating microbial endotoxins, and they produced higher levels of pro- and anti-inflammatory cytokines. 70 Fetal brain tissues from elective terminations that had experienced PAE exhibited higher expression of cytokines and chemokines, including monocyte chemoattractant protein 1 (MCP-1) and tumor necrosis factor alpha (TNF-alpha). 71 Elevated maternal cytokines were associated with worsened neurobehavioral outcomes of infants after birth. 72,73 Moreover, plasma from infants with PAE was enriched in microRNAs that target inflammatory pathways. 74 This proinflammatory state persisted postnatally and was associated with immune impairments, cognitive deficits, and greater risk for inflammation-related chronic disease in later life. 75–77

The mechanistic origins of alcohol’s proinflammatory actions outside of pregnancy are well understood. Alcohol enhances gut permeability by downregulating annexins, adherons, and other proteins that form the tight-junctions between the cells lining the gut (i.e., intestinal enterocytes and colonocytes). 78,79 Loss of this barrier permits cell wall fragments from resident Gram-negative bacteria to enter the bloodstream, and these endotoxins circulate systemically to activate host defenses through TLR and related receptors. 50 The microbial origin of this inflammation was shown by targeted removal of the Gram-negative population using bacteriophages; this intervention profoundly mitigated the hepatic inflammation of patients with alcohol-associated liver disease. 51 Additionally, alcohol may also activate the innate immune system directly by stimulating the rapid translocation and activation of TLR2 and TLR4, as shown for microglia and astrocytes. 80,81

It is likely that similar mechanisms contribute to the inflammation associated with PAE, given that a single acute alcohol exposure could increase circulating endotoxins in the mother and placenta, and perhaps in the fetus. 50 In a mouse model of PAE, microbial metabolites entered the maternal circulation and crossed the placenta to enter fetal tissues, including the brain. 63 Direct evidence that this inflammation contributes to the pathologies and behavioral deficits of PAE emerged from studies of animals lacking key proinflammatory effectors. For example, a null-mutation that inactivated the endotoxin receptor TLR4 abrogated the PAE-induced changes in plasma and brain cytokines, microglial activation, and cortical expression of synaptic and myelin-related proteins. 82,83 It also normalized offspring performance in behavioral measures reflecting anxiety, learning and memory, and social interactions. 82,83 Unexpectedly, the TLR4 null mutation also normalized neonatal body weight, perhaps reflecting the procatabolic effects of inflammation on protein metabolism. These data suggest that similar to alcohol-associated liver disease, some of the brain- and growth-related pathologies of PAE may be mediated by endotoxin and/or TLR4. Studies with germ-free animals will be key to test this potential mechanism. The maternal gut microbiome makes critical contributions to the education and maturation of the fetal immune system, 54 and alcohol’s disturbances of the maternal microbiota could also mediate persistent alterations in the offspring’s immune function.

Studies in rodents that restricted alcohol exposure to the early postnatal period (i.e., the equivalent to third-trimester brain development in humans), have suggested that inflammatory responses within the offspring also contribute to adverse effects of PAE. Coadministration of the nonsteroidal anti-inflammatory drug ibuprofen with the alcohol exposure attenuated the alcohol-induced neuroinflammation and memory deficits, 84 whereas loss of the chemokine MCP-1 or its receptor (C-C chemokine receptor type 2 [CCR2]) attenuated TLR4 activation and neuronal apoptosis. 85

However, although anti-inflammatory interventions may be efficacious, they might not operate via the proposed mechanism. For example, although many compounds have purported anti-inflammatory activities, some of these (i.e., peroxisome proliferator-activated receptor gamma [PPAR-gamma] agonists, antibiotics, phytochemicals, antioxidants) have pleiotropic actions on multiple physiological processes distinct from immune function. For example, PPAR-gamma agonists and metformin have potent metabolic actions that may separately benefit maternal and fetal development (see below), and antibiotics such as minocycline and rifampicin profoundly alter the gut microbiota, as can oral phytochemicals. 86–88 Thus, efforts to clarify the inflammatory contributions to FASD would benefit from interventions that selectively target immune functions, such as genetic knockouts and nonsteroidal anti-inflammatory drugs. A second open question is the extent to which maternal versus fetal inflammation contributes to alcohol’s pathology. Investigations that separately manipulate toll-like or cytokine receptors by genetic means in mother or fetus could clarify where an intervention is best directed. Similarly, PAE causes both systemic and brain-specific inflammation in the offspring, and the relationship, if any, between their respective responses is unclear. Tissue-specific knockouts of toll-like or cytokine/chemokine receptors would again inform their contributions. Thus, how alcohol initiates inflammation in the maternal–fetal dyad, and the actual mechanisms by which this inflammation affects the pregnancy and especially fetal brain development, remain elusive but solvable questions.

### Epigenetics, Paternal Effects, and Placental Function

#### Epigenetics mechanisms underlying effects of PAE

Similarly intertwined in PAE are the mechanistic contributions of epigenetic repatterning, including the influences of paternal alcohol exposure and their interactions with placental function. Epigenetics refers to mechanisms that impose long-term changes in gene expression without altering the DNA sequence; they typically come into play as cell fates become “locked-in” during development. 89 This is achieved through three mechanisms: DNA methylation, chromatin silencing via histone proteins, and the expression of noncoding RNAs (ncRNA). DNA methylation targets select cytosine-guanine nucleotide pairs (i.e., MeCpG) within regulatory DNA sequences to silence a gene’s expression. Long-term silencing is achieved by wrapping this methylated DNA around histone proteins. The addition or removal of methyl groups to select lysine groups within the histone regulates the degree of DNA wrapping and thus controls the gene’s expression. Further fine-tuning comes from the expression of antisense ncRNAs that bind mRNAs to prevent their translation. Elegant studies have shown that PAE alters ncRNA expression in the mother and offspring (for reviews, see Pinson and Miranda, 38 Mahnke et al., 74 and Pinson et al. 90 ). The epigenetic code or imprint is erased in the zygote and rewritten thereafter to promote in-utero survival in response to stress. 89 It also is used to silence alleles of either paternal or maternal origin as a mechanism to control fetal growth in response to in-utero stress. 89 An individual’s epigenetic signature is thought to reflect those prenatal experiences. The strongest evidence that alcohol impacts epigenetic mechanisms affecting the fetus comes from demonstrations that heavy alcohol exposure of either mother 91,92 or father 16 (see below) even before conception caused growth and physiological changes in the offspring akin to those associated with PAE. For example, preconceptual maternal alcohol exposure caused methylation-mediated silencing of the imprinted Agouti viable yellow (*A(vy)*) locus in mouse offspring. 92 Maternal stress, which is often concurrent with heavy alcohol use, was shown to be an independent influence and synergized with PAE to worsen the latter’s effects. 93,94

A large literature has documented many epigenetic responses to PAE (see Wallén et al. 95 and Gutherz et al. 17 for recent reviews). Both preclinical and clinical studies found global MeCpG differences, with more than 80% reporting hypomethylation with PAE. 96–99 However, the precise gene targets vary widely, perhaps due to differences in dosing and timing of PAE, as well as tissue and model studied. The most consistent evidence has emerged from studies of the *IGF2/H19* locus, which encodes the adjacent genes insulin-like growth factor-2 (*IGF2*) and the ncRNA *H19*. Under normal conditions, the placenta and embryo express the paternal allele of *IGF2* and maternal allele of *H19*. 100 Both are primary effectors of prenatal growth, and methylation of the paternal-derived alleles maintains their correct parent-of-origin expression in a complex regulatory manner. 100 PAE was associated with hypomethylation of the paternal *Igf2* allele in mouse placentae but not fetuses, 101 perhaps reflecting the placenta’s importance in promoting fetal growth. In adult mice with PAE, the maternal allele evidenced a hypermethylation suggestive of dysregulated imprinting. 102 A trend to hypomethylation of paternal *IGF2/H19* was also found in buccal swabs from children diagnosed with FASD 103 and in placentae from alcohol-exposed pregnancies; 104 in the latter study, the level of *IGF2/H19* hypomethylation weakly correlated with IGF2 expression and infant head circumference. In another study that did not examine the imprinted state of *IGF2*, placentae from alcohol-exposed pregnancies had increases in IGF2 expression that negatively associated with postnatal infant length and weight. 105 *IGF2/H19* showed a trend for hypomethylation in the sperm of men who drank alcohol. 106 However, hypomethylation was not found in binge-exposed male mice, nor were the methylation patterns altered in any imprinted genes of animals sired by those male mice. 107,108 Other studies of children with a history of PAE reported normal methylation at *IGF2*/*H19* but hypomethylation at several other genes, including the differentially methylated region 1 (*KvDMR1*) within the potassium voltage-gated channel *KCNQ1*, paternally expressed gene 3 (*PEG3*), and developmental pluripotency associated 4 (*DPPA4*); this latter gene has been implicated in epigenetic silencing and could inform a mechanism of alcohol’s action. 96,97 However, in all these studies the magnitude of alcohol’s effect upon MeCpG was quite small (1% to 7%) and almost never achieved 20%. Moreover, studies seldom validated the functional significance of these methylation differences by showing expression-level change for the respective genes. Indeed, a recent systematic review concluded that there was insufficient evidence associating PAE with global DNA methylation changes, hypomethylation at *IGF2/H19*, or altered methylation at other genes. 109 This raises the question whether these incremental differences in DNA methylation are functionally meaningful.

An alternate mechanism for PAE-associated epigenetic changes is alcohol’s ability to alter the epigenetic marks on histone proteins and thereby modulate chromatin structure and gene expression. This is achieved by histone methyltransferase and demethylase enzymes, with the impact on expression depending on the specific lysine or arginine residues within the histone that are targeted. PAE causes both global and gene-specific alterations in histone methylation, 17,95 although as with DNA methylation, there is no single “alcohol response.” The mechanistic evidence regarding the role of histone methylation in PAE-associated epigenetic changes is stronger than with DNA methylation. For example, the histone methyltransferase G9a generates the inactivating mark H3K9—that is, it methylates histone H3 at the lysine residue at position nine (K9) to create H3K9me2, which inhibits gene expression. Inhibition of G9a before alcohol exposure normalized its histone methyl marks and prevented alcohol-mediated cortical neuron apoptosis and behavioral deficits. 110–112 Alcohol-associated changes in chromatin structure were linked also to altered expression of developmentally important genes, including early growth response protein 1 (*Egr1*), activity-regulated cytoskeleton-associated protein (*Arc*), and Snail family transcriptional repressor 1 (*Snai1*), as well as the inflammatory genes *Tnf*, interleukin-6 (*Il6*), and others. 113–116 These latter findings suggest that epigenetic shifts may reinforce the chronic inflammation associated with PAE. Administration of a lysine dimethyltransferase inhibitor before alcohol exposure normalized the inactivating marker H3K9me2 in fetal cortex and hippocampus and improved behavior outcomes. 112 PAE also slowed histone synthesis and lengthened histone half-life, 117 perhaps reflecting alcohol’s ability to extend the cell cycle and reduce proliferation. 118

However, it is not always clear whether such differences actually reflect functional mechanisms. In both neural stem cells and mouse embryos, alcohol exposure altered histone modification, with consistent enrichment of the H3K9me2 mark that signified gene silencing; however, these chromatin-level changes did not correspond with the actual gene expressions. 119–121 Alcohol’s effects on chromatin structure in these models were gene- and dose-specific, bidirectional, and nonlinear. Nonetheless, the chromatin structure of five key developmental genes (distal-less homeobox 2 [*Dlx2*], homeobox genes A6 und A7 [*HoxA6* and *HoxA7*], Msh homeobox 2 [*Msx2*],and vitamin D receptor [*Vdr*]) was altered in both neural stem cells and the fetal cortex, and these changes persisted and stabilized over time. 121 These studies again highlight the importance of linking descriptions of chromatin remodeling to functional demonstrations of altered gene expression. Alcohol’s actions may be strongest when the exposure aligns with the gene’s window of susceptibility to epigenetic remodeling, which is a direct function of the affected cell’s pluripotency state and trajectory of differentiation. 122

Also unclear is the mechanism(s) by which PAE alters DNA and histone methylation marks. Numerous studies have reported that alcohol affected the expression or activity of epigenetic effectors, including the DNA methyltransferases (*DNMT1–4*) that create MeCpG, the methyl-CpG binding protein-2 (*MECP2*) that modulates chromatin remodeling, and the numerous histone methylases and demethylases that govern histone–DNA interactions. 121,123–125 However, these alcohol-associated differences varied with sex, tissue, and exposure model, and again there was no single “alcohol response.” Moreover, these expression-level differences did not always correlate with actual enzyme activities. 119–121 One study found persistent changes in the chromatin structure of genes that mediate chromatin remodeling, suggesting a mechanism by which alcohol could have a lasting impact on these processes. 120

Cellular metabolism also strongly affects the epigenetic methylation code. DNA and histone methyltransferases obtain their methyl groups from the metabolite *S*-adenosylmethionine (SAM) and generate *S*-adenosylhomocysteine (SAH) as an end product. SAH is a potent inhibitor of these methyltransferases, and the ratio of SAM/SAH rather than the absolute SAM content dictates the enzymes’ activity. 126 Thus, both SAM and SAH need to be measured because values for just SAM are not interpretable. SAM is regenerated from SAH through addition of methyl groups provided by choline or methionine, which may partly explain how supplemental choline attenuates alcohol’s neurobehavioral deficits. 32 It is unknown how alcohol affects methyl group availability; a recent study suggested that alcohol redirects choline fates away from SAM. 127 PAE may also limit the availability of another crucial methyl donor, folate, 33 and providing folate may further benefit choline interventions. 128

Given the current paucity of mechanistic insight into the alcohol-induced epigenetic differences, their immediate value may be as diagnostic biosignatures for PAE, assuming that the changes persist postnatally. Lussier et al. identified 299 methylated cytosine (MeC) sites in weanling rats that were shared between blood cells and the hippocampus. 129 They also identified a MeCpG profile within the buccal cells of people with FASD. 130 When tested on an independent cohort, 25% (161 of 648) of these sites again distinguished individuals with FASD from controls and did not predict autism diagnosis, sex, age, or ethnicity, suggesting their potential as a diagnostic biomarker for FASD. Analyses of whole blood from individuals with severe FASD identified six genes with both altered methylation and expression. 131 In contrast, no PAE-specific MeCpGs could be identified within the placentae or buccal swabs from offspring who experienced binge-level PAE in an Australian cohort, 132 nor in placentae, buccal swabs, and umbilical leukocytes in a South African cohort. 97 Given the millions of MeCpG sites within the human epigenome, future studies may benefit from machine learning approaches plus a consolidation of smaller datasets to facilitate biosignature discovery. 133

#### Mechanisms of paternal preconceptual alcohol exposure

Alcohol’s epigenetic actions likely also explain how paternal alcohol consumption impacts the conceptus. As reviewed elsewhere, 134 paternal drinking is associated with spontaneous abortion; 135,136 reduced birth weight and premature birth; 137 and increased risk for cardiac defects, 138 microencephaly, 139 and birth defects generally. 140 However, because drinking by a male partner facilitates maternal drinking, 134 it is difficult to disentangle alcohol’s direct effects from the socioeconomic and familial influences that surround drinking. 134,141,142

Animal studies have confirmed these paternal influences and have demonstrated reduced fetal body weight, brain weights, and placental efficiency; 107,143,144 dose-dependent craniofacial asymmetries; 145,146 and hepatic fibrosis. 147 Paternal alcohol consumption also elevated markers of cellular senescence, 148 a pathological state in which the cell loses its proliferative ability and releases proinflammatory signals. 149 Some of these paternal effects were sex-dimorphic, with male offspring exhibiting reduced fat mass, lower fasting glucose and insulin, and better glucose tolerance in response to high-fat diets. 108,147,148 These metabolic changes might reflect elevated hepatic activity of the lipogenic transcription factor LXR-alpha. 147 Behaviorally, paternal exposure was associated with increased motor activity, worsened balance and coordination, and altered alcohol-related behaviors in the offspring. 150–152

Mechanisms by which the sperm could impart its epigenetic influences include DNA damage, DNA methylation, methyl-histone modifications, and altered expression of ncRNAs. 16 Heavy alcohol consumption correlated with the hypomethylation of sperm DNA at two distinct loci, the aforementioned *H19*, and the intergenic differentially methylated region (IG-DMR) that controls paternal allele expression of genes, including iodothyronine deiodinase type-III (*DIO3*) and delta-like homolog 1 (*DLK1*). 106 However, in three separate mouse studies, the alcohol-associated sperm methylation profiles did not correlate with the offsprings’ methylation profiles or expression of paternal-imprinted genes. 107,108,153 Indeed, gamete methylation patterns are typically rewritten postimplantation and presumably this would limit such persistence. 89

Paternal alcohol exposure was instead associated with shifts in the activating histone H3K4me3 methylation mark in mouse sperm, and these correlated with placental localization of the CCCTC-binding factor (CTCF) protein that binds chromatin to regulate DNA structure and thereby gene expression. 154 However, sperm delivers not just DNA but ncRNAs, and chronic alcohol exposure has been shown to alter the ncRNA composition within sperm, including changes in tRNA-derived ncRNAs, mitochondrial ncRNAs, and microRNAs (miRNAs). 155,156 This includes selective changes in miRNAs miR-125a, miR-196a, and miR-10a/b that regulate the expression of ligand-dependent nuclear receptor corepressor (Lcor), which modulates steroid receptor interactions, and in miR-30a, miR-142, and miR-196a that regulate expression of nuclear factor erythroid 2-related factor 2 (NRF2), which controls cellular antioxidant responses. 155–157 Some miRNA changes persisted for 30 days after alcohol exposure. 157 Whether these ncRNA changes produce expression-level differences in the gamete, early embryo, or yolk sac warrants further investigation.

In summary, paternal alcohol exposure influences the conceptus, perhaps via ncRNAs and histone dysregulation, and this merits further investigation. Given that both biological parents may have heavy alcohol consumption, future work also may consider how their respective exposures interact or even synergize to shape fetal and placental development.

#### Placenta dysfunction associated with PAE

The placenta is more than a passive transporter of nutrients and gasses; it actively communicates with mother and fetus via hormones to coordinate their respective growth and adjusts those signals in response to stressors and in a sex-specific manner. 158 Its development and activity are also governed by paternal-derived epigenetic influences that further affect fetal growth. 16 The placenta arises from an embryo-derived structure, the visceral yolk sac; following implantation, it rapidly grows and differentiates to support the pregnancy. Alcohol adversely affects all these processes to increase the risk for intrauterine growth restriction, preeclampsia, and premature birth.

Whereas heavy maternal binge drinking, defined as eight standard drinks on 2 to 3 days per week, has been shown to reduce both placental and fetal growth, 159 lower-exposure levels were associated with normal or even enlarged placentae relative to fetal weight. 160 This adaptive response affected placental efficiency in a sex-dependent manner to reduce placental weight in females but not males. 160 A similar J-shaped growth response also was observed in response to paternal alcohol exposure, suggesting an epigenetic mechanism that shapes fetal growth as further discussed below. 161 Further supporting a role for epigenetic mechanisms in mediating alcohol’s effects were demonstrations that supplementation of the micronutrient choline, a methyl group donor, improved fetal growth and placental efficiency after PAE. 162,163 These epigenetic mechanisms also can redirect placental function in response to nonalcohol stressors 164 and could contribute to the effects of both pre- and postconceptual alcohol exposure and to its sex-specific effects.

Alcohol also suppressed vascularization of the visceral yolk sac 165 and placenta. 166 An embryo-derived cell lineage called trophoblasts mediates implantation into the uterine wall, and alcohol impaired trophoblast migration, invasion, and expansion, in part by invoking a temporary increase in intracellular calcium concentrations (calcium transient) that initiated their apoptosis. 167,168 Gestational alcohol use was also associated with elevated blood levels of maternal-derived miRNAs that inhibit the genes that initiate trophoblast invasion, a process called the epithelial-to-mesenchymal transition (EMT). The addition of this miRNA “cocktail” was sufficient to inhibit the EMT and proliferative expansion of cultured trophoblasts and reduced placental and fetal growth in vivo. 90,169 Alcohol-mediated reductions in IGF1 signaling and activity may further limit placenta formation. 170,171

Alcohol-exposed placentae exhibit morphological and vascularity abnormalities, including uteroplacental malperfusion (i.e., inadequate blood supply to the uterus and placenta), impaired vascular remodeling, and chorangiosis (i.e., an excess of capillaries in the placenta that may indicate hypoxia). 170,172,173 This is accompanied by vascular dysfunction with an exaggerated vasoconstriction response to hormones, such as angiotensin II. 174–176 The subsequent reduction of placental blood flow limits the transport of nutrients, such as glucose, 177,178 amino acids 179 and folate, 180 and causes fetal hypoxia. 175,176,181 Reductions in the vasodilator endothelial nitric oxide synthase (eNOS) 176 and increased reactivity to the vasoconstrictive hormone thromboxane B2 182 may underlie this vasoconstriction; supplementation with phosphatidic acid normalizes eNOS and vascular tone in the uterine artery. 183 Preclinical models revealed that alcohol suppressed the expression of key genes that promote blood vessel formation (angiogenesis), including vascular endothelial growth factor (VEGF) and its receptor, kinase insert domain receptor (KDR). 166,184 Similar findings were reported for placentae from women who drank heavily in pregnancy, with dysregulated expression of angiogenic genes, including annexin-A4, KDR, scavenger receptor class B type 1 (SCARB1), ETS proto-oncogene 1 (ETS1), and EGL nine homolog 1 (EGLN1). 185,186 Additionally, recent work has identified a placenta–brain axis in which placental growth factor (PlGF) was released into the fetal circulation to enhance VEGF activity and stimulate the brain’s vascular development. 187 PAE reduced the placental production of both PlGF 188 and a second interacting protein, CD146. 189 Moreover, placental knockdown of either PlGF or CD146 caused vascular deficits in the brain cortex that were similar to those from PAE. 190 Conversely, PlGF overexpression attenuated both the placental and cortical derangements due to PAE, suggesting it may be a candidate for intervention. 188,191 Comparative genomics of the fetal placenta and cortex with and without PAE identified an expression signature that featured genes related to angiogenesis and vascular development, and both organs exhibited a specific loss of angiotensin protein but not its receptors. 191 This altered cortical vascularity persists into adulthood and may exacerbate responses to stroke in later life. 192 There is interest in whether placental vascularity may be a surrogate marker for PAE (for additional discussions of PAE and placental vascularity, see Gualdoni et al. 166).

Alcohol’s proinflammatory actions also extend to the placenta, which exhibits a proinflammatory gene expression profile and potential enrichments in Hofbauer cells, which are fetal-derived macrophages that may recruit maternal T cells to the chorionic villi. 193,194 The gene expression profile included elevations in cytokines (interleukin [IL] 1-alpha, IL-1-beta, IL-6, interferon gamma [IFN-gamma], TNF-alpha, TNF superfamily member 4 [TNFsf4]) and chemokine mediators (C-C motif chemokine ligand 20 [CCL20], CCR2, CCR1). 185,194–196 In other instances, chronic placental inflammation was linked to villus destruction, reduced nutrient transfer (especially for iron, see below), fetal growth restriction, neurocognitive deficits, and chronic disease in later life. 197,198 The consequences of this placental inflammation for placental dysfunction in PAE are not well studied and merit further investigation. Although the placenta itself is likely sterile, 199 alcohol might compromise its impermeability to pathogens and endotoxins to promote a chronic proinflammatory state.

In summary, renewed attention to alcohol’s impact on the placenta has created a strong foundation for future investigations. Additional opportunities include alcohol’s impact on placental metabolic and transport roles that support the fetus. Nutrients do not simply “flow” from mother to fetus, and their bidirectional movement is tightly regulated. The placenta achieves this via a metabolic governance that is distinct from that of the mother and fetus. 200 Alcohol’s impact here is essentially unknown.

### Metabolic Derangements and Functional Iron Deficiency

#### Metabolic mechanisms underlying the effects of PAE

Alcohol has multiple metabolic effects as a nutrient-poor caloric source that is converted to acetaldehyde, acetate, ketones, fats, and ultimately CO_2_. Its metabolic consequences are well understood in the nonpregnancy state and include hepatic steatosis and peripheral lipid mobilization, skeletal muscle atrophy (sarcopenia), greater insulin resistance, ketosis, sharply elevated ratios of reduced to oxidized forms of nicotinamide adenine dinucleotide (NADH/NAD), mitochondrial dysfunction, and the potential displacement of essential nutrients. 7,201 Limited attention has been paid to these alcohol-responsive processes in pregnancy, during which the mother undergoes extensive metabolic remodeling to support the fetus’s extraordinary anabolic state. Disruptions in maternal metabolism have cascading fetal consequences, including preeclampsia, gestational diabetes, and fetal growth restriction. 158,202 Recent preclinical evidence has suggested similar disruptions in alcohol-exposed pregnancies. 177,178 However, it should be cautioned that some of these metabolic changes may be adaptive rather than pathological.

Acute alcohol exposure has been shown to reduce maternal plasma glucose. 177,178,203,204 Untargeted metabolomics of maternal mouse liver revealed that this is accompanied by reductions in key glycolytic intermediates but not tricarboxylic acid (TCA) cycle intermediates, suggesting reduced glycolytic flux. 204 The fetal brains and placentae from the alcohol-exposed pregnancies also had reduced glucose levels, whereas the fetal livers made a compensatory attempt to increase glucose by activating gluconeogenesis and increasing expression of key enzymes glucose-6-phosphatase and phosphoenolpyruvate carboxykinase (PEPCK). 177 Fetal urea and amino acid catabolites also rose as animo acids were diverted into gluconeogenesis at the expense of protein synthesis, and these elevated catabolites and gluconeogenic enzymes negatively correlated with fetal growth. 177,204 Potentially exacerbating these glucose losses was an increased glycogen deposition within PAE placentae 163 and a diversion of placental glucose into pentose phosphate pathway intermediates and glucosamine synthesis. 178

During a healthy pregnancy, the mother acquires a partial insulin resistance that makes more plasma glucose available for placental and fetal use while the maternal metabolism emphasizes lipids as a major fuel. 202 PAE prevented this adaptation, and alcohol-exposed mouse dams retained their insulin sensitivity as reflected in their rapid clearance of plasma glucose following administration of insulin or a glucose bolus. 177 However, they also exhibited normal pancreatic insulin release and blunted hepatic insulin signaling. Thus, the underlying mechanism preventing their acquisition of insulin resistance remains unclear.

Insight may emerge from preclinical studies in which coadministration of the TORC1 inhibitor rapamycin and alcohol attenuated the offspring’s learning and memory deficits; 205 TORC1 is a primary effector of cellular anabolism. This study targeted neonatal pups; therefore, whether maternal metabolism might also benefit is unknown. Although oxidative stress measures were also reduced in the pups, TORC1 inhibition has diverse impacts and most notably the activation of adenosine monophosphate-activated protein kinase (AMPK), which promotes adaptive metabolic changes in response to stress. 206 Thus, it is unclear how rapamycin countered alcohol’s actions.

With respect to lipids, both clinical and preclinical studies have documented altered lipid profiles in alcohol-exposed mothers and fetuses, with consistent elevations in free fatty acids, glycerides, and phospholipids derived from palmitate (C16:0), stearate (C18:0), and linoleate (C18:2). 61,65,204,207 Circulating very low density and low density lipoproteins (VLDL and LDL) were reduced during pregnancy in women who drank heavily 208 as were individual phospholipids and triglycerides in a rat PAE model. 207 These changes likely reflect alcohol’s mobilization of free fatty acids from adipocyte stores, reduced hepatic VLDL synthesis, and increased hepatic lipid synthesis, elongation, and desaturation. 201 Maternal plasma lipids rise during a healthy pregnancy, which provides the mother with an alternate energy source and spares her glucose for fetal use. 158 The alcohol-driven reductions in plasma lipoproteins could limit maternal energy availability and might be driven by the same signals that blunt the mother’s gestational insulin resistance. Potentially supporting this is the recent demonstration that maternal supplementation with phosphoglycerate normalized fetal growth in a rat PAE model. 183 Although the authors posited that phosphoglycerate acts by enhancing uterine artery relaxation, the compound is also the backbone on which phospholipids are built and could be limiting in a highly lipogenic environment. Another study in a rat PAE model reported that the postnatal brain might accumulate lipid droplets, 61 and this could be maladaptive or perhaps adaptive to prior reductions in fetal brain glucose. 177

Amino acid pools are tightly regulated and alcohol has different effects on these, depending on the compartment (maternal versus fetal), tissue, and exposure model. 203,204,209 For example, chronic alcohol use reduced essential amino acid levels in maternal liver 204 but elevated most amino acids in fetal brain hippocampus and cerebellum. 210 Untargeted metabolomics studies have demonstrated that elevations in amino acid catabolites can distinguish alcohol-exposed pregnancies. For example, a study in pregnant women (18.5 ± 6.5 weeks gestation) who drank heavily found elevated blood glutamate and glycine levels, both of which are critical for nucleoside synthesis; blood glutamate was negatively associated with infant length and weight, and glycine was negatively associated with infant weight and head circumference. 65 Similarly, in a mouse model of PAE, amino acid catabolites, including urea, kynurenate, and citrulline, were elevated and negatively correlated with fetal brain and body weight. 204 The rise in these catabolites may reflect the aforementioned fetal need to generate glucose via gluconeogenesis. Supporting this is the alleviation of fetal growth restriction by glutamine infusion in an ovine PAE model. 203,211 Glutamine, in addition to protein synthesis, also supplies carbon skeletons for the TCA cycle and nitrogen for nucleoside synthesis.

In summary, alcohol’s metabolic effects are likely to be a major driver of PAE-associated pathologies during the heavily anabolic pregnancy. Moreover, many at-risk pregnancies involve women who exhibited heavy drinking patterns before pregnancy, and it is unknown how the resulting metabolic changes before pregnancy affect the mother’s ability to adapt to and support a healthy pregnancy. The extrapolation of alcohol’s known impacts in a nonpregnant state to PAE should greatly enhance understanding of the mechanisms underlying PAE’s effects on the fetus and promote the design of macronutrient and/or pharmacological interventions that could address those metabolic changes. Additionally, PAE-related metabolic changes may contribute to alcohol’s epigenetic reprogramming, both by creating maternal and fetal nutrient deficiencies that induce fetal stress, and by altering methyl or acetyl availability as discussed above. It is worth noting that alcohol is a molar-level source of acetate groups (one U.S. standard drink of 14 g ethanol contains 0.3 mol acetate) and may modulate the availability of acetate groups used for histone modification.

#### Functional iron deficiency and the anemia associated with PAE

Intertwined with many of the mechanisms contributing to the effects of PAE—impact on microbiome, inflammation, placental function, and metabolism—is alcohol’s impairment of the erythrocytic and vascular expansion necessary to support fetal growth. Formation of red blood cells (erythropoiesis) originates in the yolk sac, then shifts to the nascent liver for much of development, before moving to bone marrow near term. 212 Growing evidence indicates that PAE is associated with fetal anemia, 213,214 which would be one of its most impactful mechanisms to impair fetal development. Fetal anemia is associated with lasting cognitive and behavioral impairments, not just due to hypoxia, but also due to the loss of iron that is critical for healthy brain development. 215 Iron is essential for myelination and adenosine triphosphate generation and catalyzes the synthesis and disposal of neuroactive amines. 215 Rodent studies revealed that PAE caused fetal anemia characterized by red blood cells with normal size but with significant declines in red cell counts, hematocrit, and hemoglobin (normocytic, hypochromic anemia). 213,216,217 A fourfold increase in iron-deficient anemia and reduced hemoglobin was also documented in infants born to mothers who reported binge drinking in a South African cohort. 214,218 The infants’ risk for iron-deficient anemia was even greater in childhood and was negatively associated with infant growth. 214,218,219 This fetal “anemia of PAE” can occur even when the mother has adequate iron levels and is not anemic; thus, her clinical indicators mask the fetus’s anemia. 213,218,220 Fetal outcomes are further worsened when the mother herself has iron deficiency, and the combination of iron deficiency and PAE further reduces placental efficiency and fetal and postnatal growth; worsens inflammation; and synergizes to reduce learning and memory. 195,221–224 The extent and severity of this fetal anemia associated with PAE is unknown, even though iron deficiency is the most common single-nutrient deficiency in pregnancy. 225

It has become clear that this fetal anemia arises, in part, because PAE creates a functional iron deficiency in the fetus—that is, maternal iron intake is adequate, but this iron is poorly utilized. 226 Preclinical studies found that PAE reduced and dysregulated iron levels in fetal brain 213,227,228 and plasma 229 because it sequesters iron within fetal hepatic stores (which with PAE contain 123% of control iron levels), making it inaccessible for other organs, including the erythrocytes and their precursors as well as the brain. 213 A similar iron sequestration has been suggested by the elevated levels of ferritin (a protein that sequesters iron) and reduced hemoglobin:ferritin ratios seen in pregnant women who drank heavily and their infants. 220,230 Functional iron deficiency was further supported by the red cells’ normocytic, hypochromic appearance and the increased numbers of immature red cells in the fetal liver sinusoids. 216

A common cause of functional iron deficiency is chronic inflammation, in which cytokines stimulate the hepatic production of hepcidin, a circulating peptide that inhibits iron absorption and promotes iron storage. 231 Hepcidin levels were elevated in pregnant women who reported binge drinking, and these elevations correlated with infant risk for iron deficiency anemia. 220 Preclinical models of PAE showed that alcohol stimulated hepcidin production through the IL6-mediated activation of signal transducer and activator of transcription 3 (Stat3) signaling. 232 The proinflammatory state of the alcohol-exposed placenta 186,194,195 may be an additional driver of the fetus’s functional iron deficiency as it coordinates maternal–fetal iron transfer 233 and promotes fetal angiogenesis and erythropoiesis, 187 signals that are also disrupted by PAE (see above). This inflammation-stimulated elevation of hepcidin may explain why the fetus’s anemic state is not normalized despite attempts to elevate these pro-angiogenic and pro-hematopoietic signals. 186,194,217 Provision of supplemental iron during PAE reversed both elevated hepcidin and anemia, suggesting that PAE increased fetal iron requirements. 216,234 This mechanistic pathway may also explain the ability of anti-inflammatories to attenuate alcohol-induced fetal damage, as these agents would reduce the cytokines that stimulate hepcidin synthesis and limit iron utilization. 69 An important question is whether mitigating inflammation during PAE would also mitigate the fetal anemia, as has been shown for the nonpregnancy state. 235 Similarly, erythropoietin agonists (e.g., roxadustat) are efficacious in treating the anemia of chronic kidney disease and might have merit as an intervention in PAE. 236

PAE may also directly suppress erythropoiesis. High rates of erythropoiesis are required to sustain the fetus’s rapid growth. However, red blood cell proliferation places a high demand on the generation of the ribosomes necessary to support that growth, such that genetic deficits in ribosome biogenesis are typified by an anemia that is refractory to supplemental iron or folate. 237 Alcohol directly impairs ribosome biogenesis in highly proliferative cells, such as neural stem cells and neural crest. 238,239 Alcohol also suppresses the anabolic effector TORC1, which otherwise induces ribosome biogenesis through its activation of ribosomal protein S6 kinase (RPS6K). 24 It is unknown if impaired ribosome biogenesis also affects fetal hepatic erythropoiesis; however, fetal mouse liver under PAE exhibited reduced expression of key genes that promote ribosome biogenesis, including treacle ribosome biogenesis factor 1 (*Tcof*). 217 Given the grave repercussions of fetal anemia, the anemia of PAE and its potential interventions warrant a high priority for study.

## Conclusions

**Figure 2:**
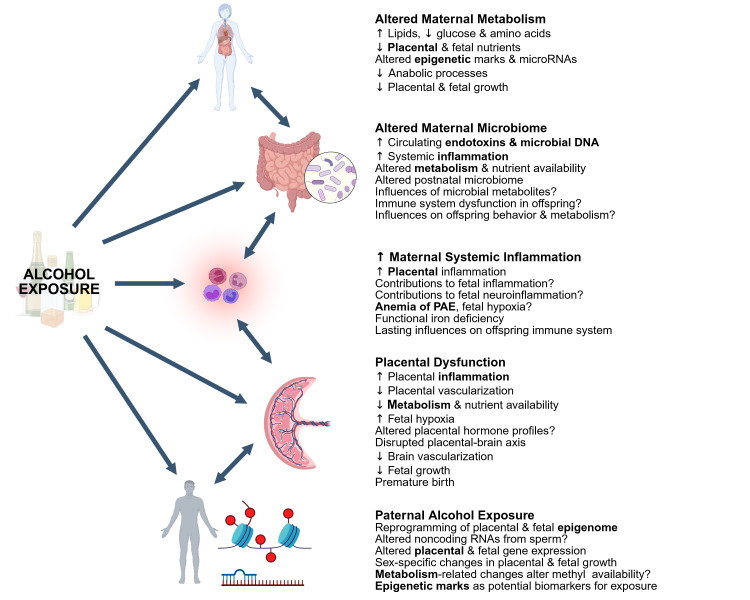
Integrative summary of nonfetal mechanisms that contribute to alcohol’s teratogenicity. In addition to its direct interactions with the conceptus, alcohol affects additional organs and compartments that have critical influences upon healthy fetal development. These include effects on the biological mother and her microbiome communities, the placenta, and the biological father. These further interact with each other, and with the conceptus, to produce systemic inflammation, vascular dysfunction, metabolic insufficiencies, fetal anemia, and epigenetic reprogramming. These effects, in turn, alter cellular signaling and metabolism to disrupt myriad processes, including cell proliferation, differentiation, migration, and survival. Boldface terms indicate where these nonfetal mechanisms interact. Figure generated in BioRender.

This review has highlighted new opportunities for research into the mechanisms underlying alcohol’s teratogenicity, focusing on factors that are largely external to the conceptus yet have critical influences upon its development. This includes several alcohol-related pathologies that are seldom considered within the framework of pregnancy. The article further emphasizes how “no mechanism is an island” and discusses how these areas are mechanistically interwoven to perhaps synergize or accelerate alcohol’s pathogenicity. These interrelationships are summarized in Figure 2 . For example, alcohol-induced alterations to enteric microbial communities may affect nutrient availability to the mother and fetus and could redirect their respective immune systems to generate short- and long-term dysfunction. Alcohol and its secondary metabolites themselves have proinflammatory actions that are further fueled by the accompanying gut—and perhaps placental—permeability that permits entry of microbial endotoxins and DNA fragments into the bloodstream. These circulate within the mother, placenta, and conceptus to sustain a chronic inflammatory state. This chronic inflammation may also contribute to the fetal anemia of PAE that limits iron availability and could promote a fetal hypoxic state that impairs its growth and brain development. Fetal growth may be further limited by alcohol-induced metabolic insufficiencies that cannot support the pregnancy’s profoundly anabolic state—changes that reflect not only potential dietary insufficiency but alcohol’s disruption of macronutrient utilization. Such metabolic disruptions could alter availability of the one-carbon and acetyl units, and perhaps also the microRNAs, involved in epigenetic modifications, not only within the uterine environment but also in the father.

Overall, this integrative view suggests that interventions likely to target foundational mechanisms with the widest range of influences may have the greatest efficacy. Similarly, highly focused interventions may have greater efficacy when provided with other interventions that address complementary targets. However, focused examinations of individual mechanisms remain critical because they inform an understanding of those mechanisms and an optimized design of interventions targeting them.

The advances described here have generated outstanding opportunities to expand understanding of alcohol’s fundamental mechanisms of action. A major limitation is that additional mechanisms likely remain undiscovered because a full description of the proteins with which alcohol directly interacts to alter their activity is still lacking. In silico approaches that incorporate structural modeling plus deep learning algorithms such as Alpha-Fold could accelerate discovery of these proteins to identify novel mechanisms and interventions. It is also unclear which of the described preclinical findings are relevant for humans. In silico modeling of target protein structures across species would inform this question, as would expanded testing across taxa to identify mechanisms that are evolutionarily conserved. Another area of opportunity is for increased collaboration between the organ-specific alcohol research communities. For example, much is known about alcohol’s mechanisms in the nonpregnant state. However, these mechanisms are seldom applied to pregnancy even though they almost certainly operate during an alcohol-exposed pregnancy. Similarly, mechanisms that act on the brain could be operational in other organs and vice versa—for example, as shown for the astrocytic metabolism of alcohol 240 and neurotransmitter actions on the gut and immune system. 241,242 An additional limitation is the frequent absence of diet information in animal studies, even though diet has a major influence on outcomes involving the microbiome, inflammation, nutrient influences, and the interplay of epigenetics and metabolism. The current Animal Research: Reporting of In Vivo Experiments (ARRIVE) Guidelines do not require reporting of diet composition or identity; such a mandate would enhance the reproducibility of preclinical studies.

Because alcohol is a pleiotropic drug that interacts with numerous proteins to perturb cellular and physiological processes, it remains a significant but solvable challenge to isolate alcohol’s underlying mechanistic actions. This mechanistic understanding is crucial to the design of interventions that specifically target and thereby remediate alcohol’s upstream actions. Without this understanding, an intervention risks becoming a bandage that stops a subset of outcomes that may or may not be relevant to alcohol’s pathologies. This review has highlighted a subset of mechanisms for which there is good biological plausibility. Increasing attention to these can significantly advance our mechanistic understanding of alcohol’s teratogenicity.

**KEY TAKEAWAYS**
- Alcohol targets not just the embryo and fetus (i.e., the conceptus) but also the mother, biological father, placenta, and maternal microbiome to further disrupt embryo–fetal development.
- Alcohol reprograms the maternal enteric microbiota and causes persistent alterations in the offspring’s microbiota, with potentially adverse consequences for the offspring’s immune system, gut mucosa, and behavior.
- Circulating endotoxins from the mother’s enteric microbiota may contribute to the inflammation associated with prenatal alcohol exposure and to disturbances in maternal nutrition and fetal immunity.
- Alcohol-mediated reprogramming of the placental and fetal epigenome may contribute to placental dysfunctions and fetal growth reductions.
- Paternal alcohol consumption may alter the epigenetic signals delivered by the sperm to further influence fetal and placental development, perhaps in a sex-specific manner.
- Disruptions of the placenta-brain axis, including reductions in placental growth factor, may contribute to the vascular, structural, and functional deficits of the developing brain.
- Alterations of maternal metabolism, including a failure to acquire gestational insulin resistance, limit fetal glucose availability and further contribute to fetal growth deficits.
- Prenatal alcohol exposure and its associated inflammation may cause a fetal anemia that combined with vascular deficits could drive a fetal hypoxia that furthers limit fetal growth and development.

## Figures and Tables

**Table 1:** Search Parameters and Results (Number of Studies) for the Narrative Review

Search Parameters	Articles Retrieved	Not Relevant	Selected	Included	Excluded
Microbiome [Ti/Ab] (1981–2024)	24	0	24	5	19
Inflammation[Ti/Ab]) OR neuroinflamm[Ti/Ab] OR inflammatory[MeSH] OR cytokine[Ti/Ab] (2000–2024)	129	37	92	72	20
Epigenetics[Ti/Ab] (2000–2024)	182	104	78	30	48
Paternal[Ti/Ab] (1981–2024)	54	10	44	34	10
Placenta[Ti/Ab] (1981–2024)	121	53	68	53	15
Metabolism[MeSH] OR lipid[Ti/Ab] OR amino acid[Ti/Ab] OR insulin[Ti/Ab] (2000–2024)	378	338	40	37	3
Iron[Ti/Ab]) OR iron deficiency[Ti/Ab] OR anemia[Ti/Ab] (1981–2024)	41	5	36	23	13

*Note:* All searches also included “fetal alcohol spectrum disorder[MeSH] OR fetal alcohol[Ti/Ab].”

## References

[1] Hoyme HE, Kalberg WO, Elliott AJ (2016). Updated clinical guidelines for diagnosing Fetal alcohol spectrum disorders. Pediatrics.

[2] Wozniak JR, Riley EP, Charness ME (2019). Clinical presentation, diagnosis, and management of fetal alcohol spectrum disorder. Lancet Neurol.

[3] May PA, Chambers CD, Kalberg WO (2018). Prevalence of fetal alcohol spectrum disorders in 4 US communities. JAMA.

[4] Gosdin LK, Deputy NP, Kim SY, Dang EP, Denny CH (2022). Alcohol consumption and binge drinking during pregnancy among adults aged 18–49 years—United States, 2018–2020. MMWR Morb Mortal Wkly Rep.

[5] Edenberg HJ (2007). The genetics of alcohol metabolism: Role of alcohol dehydrogenase and aldehyde dehydrogenase variants. Alcohol Res Health.

[6] Wu D, Cederbaum AI (2003). Alcohol, oxidative stress, and free radical damage. Alcohol Res Health.

[7] Simon L, Souza-Smith FM, Molina PE (2022). Alcohol-associated tissue injury: Current views on pathophysiological mechanisms. Annu Rev Physiol.

[8] Lu Y, George J (2024). Interaction between fatty acid oxidation and ethanol metabolism in liver. Am J Physiol Gastrointest Liver Physiol.

[9] Mukerjee S, Siciliano CA (2025). Richardson’s law and the origins of alcohol research. Proc Natl Acad Sci USA.

[10] Mihic SJ, Ye Q, Wick MJ (1997). Sites of alcohol and volatile anaesthetic action on GABA_A_ and glycine receptors. Nature.

[11] Dwyer DS, Bradley RJ (2000). Chemical properties of alcohols and their protein binding sites. Cell Mol Life Sci.

[12] Aryal P, Dvir H, Choe S, Slesinger PA (2009). A discrete alcohol pocket involved in GIRK channel activation. Nat Neurosci.

[13] Chen SY, Wilkemeyer MF, Sulik KK, Charness ME (2001). Octanol antagonism of ethanol teratogenesis. FASEB J.

[14] Garic-Stankovic A, Hernandez M, Flentke GR, Smith SM (2006). Structural constraints for alcohol-stimulated Ca2+ release in neural crest, and dual agonist/antagonist properties of *n*-octanol. Alcohol Clin Exp Res.

[15] Jumper J, Evans R, Pritzel A (2021). Highly accurate protein structure prediction with AlphaFold. Nature.

[16] Golding MC (2023). Teratogenesis and the epigenetic programming of congenital defects: Why paternal exposures matter. Birth Defects Res.

[17] Gutherz OR, Deyssenroth M, Li Q (2022). Potential roles of imprinted genes in the teratogenic effects of alcohol on the placenta, somatic growth, and the developing brain. Exp Neurol.

[18] Upreti D, Rouzer SK, Bowring A (2023). Microbiota and nutrition as risk and resiliency factors following prenatal alcohol exposure. Front Neurosci.

[19] Zuo Y, Aistrup GL, Marszalec W (2001). Dual action of *n-*alcohols on neuronal nicotinic acetylcholine receptors. Mol Pharmacol.

[20] Luo J (2014). Autophagy and ethanol neurotoxicity. Autophagy.

[21] Tucker SK, Eberhart JK (2024). The convergence of mTOR signaling and ethanol teratogenesis. Reprod Toxicol.

[22] Carabulea AL, Janeski JD, Naik VD, Chen K, Mor G, Ramadoss J (2023). A multi-organ analysis of the role of mTOR in fetal alcohol spectrum disorders. FASEB J.

[23] Martín-Estal I, Castilla-Cortázar I, Castorena-Torres F (2021). The placenta as a target for alcohol during pregnancy: The close relation with IGFs signaling pathway. Rev Physiol Biochem Pharmacol.

[24] Huang Y, Flentke GR, Smith SM (2024). Alcohol induces p53-mediated apoptosis in neural crest by stimulating an AMPK-mediated suppression of TORC1, S6K, and ribosomal biogenesis. Reprod Toxicol.

[25] Boschen KE, Fish EW, Parnell SE (2021). Prenatal alcohol exposure disrupts Sonic hedgehog pathway and primary cilia genes in the mouse neural tube. Reprod Toxicol.

[26] Serio RN, Gudas LJ (2020). Modification of stem cell states by alcohol and acetaldehyde. Chem Biol Interact.

[27] Siggins RW, McTernan PM, Simon L, Souza-Smith FM, Molina PE (2023). Mitochondrial dysfunction: At the nexus between alcohol-associated immunometabolic dysregulation and tissue injury. Int J Mol Sci.

[28] Terracina S, Tarani L, Ceccanti M (2024). The impact of oxidative stress on the epigenetics of fetal alcohol spectrum disorders. Antioxidants.

[29] Das U, Gangisetty O, Chaudhary S (2023). Epigenetic insight into effects of prenatal alcohol exposure on stress axis development: Systematic review with meta-analytic approaches. Alcohol Clin Exp Res.

[30] Ruffaner-Hanson C, Noor S, Sun MS (2022). The maternal-placental-fetal interface: Adaptations of the HPA axis and immune mediators following maternal stress and prenatal alcohol exposure. Exp Neurol.

[31] Naik VD, Lee J, Wu G, Washburn S, Ramadoss J (2022). Effects of nutrition and gestational alcohol consumption on fetal growth and development. Nutr Rev.

[32] Ernst AM, Gimbel BA, de Water E (2022). Prenatal and postnatal choline supplementation in fetal alcohol spectrum disorder. Nutrients.

[33] Steane SE, Cuffe JSM, Moritz KM (2023). The role of maternal choline, folate and one-carbon metabolism in mediating the impact of prenatal alcohol exposure on placental and fetal development. J Physiol.

[34] Helfrich KK, Saini N, Kling PJ, Smith SM (2018). Maternal iron nutriture as a critical modulator of fetal alcohol spectrum disorder risk in alcohol-exposed pregnancies. Biochem Cell Biol.

[35] Sambo D, Goldman D (2023). Genetic influences on Fetal alcohol Spectrum Disorder. Genes.

[36] Smith SM, Weathers TD, Virdee MS (2024). Polymorphisms in the choline transporter *SLC44A1* are associated with reduced cognitive performance in normotypic but not prenatal alcohol-exposed children. Am J Clin Nutr.

[37] Pinson MR, Chung DD, Adams AM (2021). Extracellular vesicles in premature aging and diseases in adulthood due to developmental exposures. Aging Dis.

[38] Pinson MR, Miranda RC (2019). Noncoding RNAs in development and teratology, with focus on effects of cannabis, cocaine, nicotine, and ethanol. Birth Defects Res.

[39] Bariselli S, Lovinger DM (2021). Corticostriatal circuit models of cognitive impairments induced by fetal exposure to alcohol. Biol Psychiatry.

[40] Leung ECH, Jain P, Michealson MA, Choi H, Ellsworth-Kopkowski A, Valenzuela CF (2024). Recent breakthroughs in understanding the cerebellum’s role in fetal alcohol spectrum disorder: A systematic review. Alcohol.

[41] Topchiy I, Mohbat J, Folorunso OO, Wang ZZ, Lazcano-Etchebarne C, Engin E (2024). GABA system as the cause and effect in early development. Neurosci Biobehav Rev.

[42] Basavarajappa BS, Subbanna S (2023). Synaptic plasticity abnormalities in fetal alcohol spectrum disorders. Cells.

[43] Licheri V, Brigman JL (2021). Altering cell-cell interaction in prenatal alcohol exposure models: Insight on cell-adhesion molecules during brain development. Front Mol Neurosci.

[44] Chen SY, Kannan M (2023). Neural crest cells and fetal alcohol spectrum disorders: Mechanisms and potential targets for prevention. Pharmacol Res.

[45] Flentke GR, Baulch JW, Berres ME, Garic A, Smith SM (2019). Alcohol-mediated calcium signals dysregulate pro-survival Snai2/PUMA/Bcl2 networks to promote p53-mediated apoptosis in avian neural crest progenitors. Birth Defects Res.

[46] Hou K, Wu ZX, Chen XY (2022). Microbiota in health and diseases. Signal Transduct Target Ther.

[47] National Institute on Alcohol Abuse and Alcoholism (2025). Understanding alcohol drinking patterns.

[48] Litwinowicz K, Choroszy M, Waszczuk E (2020). Changes in the composition of the human intestinal microbiome in alcohol use disorder: A systematic review. Am J Drug Alcohol Abuse.

[49] Gregory AL, Pensinger DA, Hryckowian AJ (2021). A short chain fatty acid-centric view of *Clostridioides difficile* pathogenesis. PLOS Pathog.

[50] Bala S, Marcos M, Gattu A, Catalano D, Szabo G (2014). Acute binge drinking increases serum endotoxin and bacterial DNA levels in healthy individuals. PLOS One.

[51] Duan Y, Llorente C, Lang S (2019). Bacteriophage targeting of gut bacterium attenuates alcoholic liver disease. Nature.

[52] Barrientos G, Ronchi F, Conrad ML (2024). Nutrition during pregnancy: Influence on the gut microbiome and fetal development. Am J Reprod Immunol.

[53] Cryan JF, O’Riordan KJ, Cowan CSM (2019). The microbiota-gut-brain axis. Physiol Rev.

[54] Gomez de Agüero M, Ganal-Vonarburg SC, Fuhrer T (2016). The maternal microbiota drives early postnatal innate immune development. Science.

[55] Zheng D, Liwinski T, Elinav E (2020). Interaction between microbiota and immunity in health and disease. Cell Res.

[56] Cryan JF, Mazmanian SK (2022). Microbiota–brain axis: Context and causality. Science.

[57] Hsiao EY, McBride SW, Hsien S (2013). Microbiota modulate behavioral and physiological abnormalities associated with neurodevelopmental disorders. Cell.

[58] Bodnar TS, Lee C, Wong A, Rubin I, Wegener Parfrey L, Weinberg J (2022). Evidence for long-lasting alterations in the fecal microbiota following prenatal alcohol exposure. Alcohol Clin Exp Res.

[59] Vella VR, Ainsworth-Cruickshank G, Luft C (2025). Dysregulation of immune system markers, gut microbiota and short-chain fatty acid production following prenatal alcohol exposure: A developmental perspective. Neurochem Int.

[60] Bodnar TS, Ainsworth-Cruickshank G, Billy V, Wegener Parfrey L, Weinberg J, Raineki C (2024). Alcohol consumption during pregnancy differentially affects the fecal microbiota of dams and offspring. Sci Rep.

[61] Hwang HM, Kawasawa YI, Basha A, Mohammad S, Ito M, Hashimoto-Torii K (2023). Fatty acid metabolism changes in association with neurobehavioral deficits in animal models of fetal alcohol spectrum disorders. Commun Biol.

[62] Nguyen TLA, Vieira-Silva S, Liston A, Raes J (2015). How informative is the mouse for human gut microbiota research?. Dis Model Mech.

[63] Virdee MS, Saini N, Kay CD (2021). An enriched biosignature of gut microbiota-dependent metabolites characterizes maternal plasma in a mouse model of fetal alcohol spectrum disorder. Sci Rep.

[64] Vuong HE, Pronovost GN, Williams DW (2020). The maternal microbiome modulates fetal neurodevelopment in mice. Nature.

[65] Hasken JM, de Vries MM, Marais AS (2022). Untargeted metabolome analysis of alcohol-exposed pregnancies reveals metabolite differences that are associated with infant birth outcomes. Nutrients.

[66] Kim D, Hofstaedter CE, Zhao C (2017). Optimizing methods and dodging pitfalls in microbiome research. Microbiome.

[67] Laukens D, Brinkman BM, Raes J, De Vos M, Vandenabeele P (2016). Heterogeneity of the gut microbiome in mice: Guidelines for optimizing experimental design. FEMS Microbiol Rev.

[68] Mukherjee S, Tarale P, Sarkar DK (2023). Neuroimmune interactions in fetal alcohol spectrum disorders: Potential therapeutic targets and intervention strategies. Cells.

[69] Kane CJM, Drew PD (2021). Neuroinflammatory contribution of microglia and astrocytes in fetal alcohol spectrum disorders. J Neurosci Res.

[70] Maxwell JR, Noor S, Pavlik N (2024). Moderate prenatal alcohol exposure increases toll-like receptor activity in umbilical cord blood at birth: A pilot study. Int J Mol Sci.

[71] Darbinian N, Darbinyan A, Merabova N (2021). Ethanol-mediated alterations in oligodendrocyte differentiation in the developing brain. Neurobiol Dis.

[72] Bodnar TS, Raineki C, Wertelecki W (2018). Altered maternal immune networks are associated with adverse child neurodevelopment: Impact of alcohol consumption during pregnancy. Brain Behav Immun.

[73] Sowell KD, Uriu-Adams JY, Van de Water J (2018). Implications of altered maternal cytokine concentrations on infant outcomes in children with prenatal alcohol exposure. Alcohol.

[74] Mahnke AH, Sideridis GD, Salem NA (2021). Infant circulating microRNAs as biomarkers of effect in fetal alcohol spectrum disorders. Sci Rep.

[75] Fiore M, Petrella C, Coriale G (2022). Markers of neuroinflammation in the serum of prepubertal children with fetal alcohol spectrum disorders. CNS Neurol Disord Drug Targets.

[76] Bodnar TS, Mak DY, Hill LA, Ellis L, Yu W, Weinberg J (2022). Modulatory role of prenatal alcohol exposure and adolescent stress on the response to arthritis challenge in adult female rats. eBioMedicine.

[77] Deak T, Kelliher KT, Wojcik HJ, Gano A (2022). Prenatal and adolescent alcohol exposure programs immunity across the lifespan: CNS-mediated regulation. Pharmacol Biochem Behav.

[78] Yang Y, Schnabl B (2024). Gut bacteria in alcohol-associated liver disease. Clin Liver Dis.

[79] Llopis M, Cassard AM, Wrzosek L (2016). Intestinal microbiota contributes to individual susceptibility to alcoholic liver disease. Gut.

[80] Fernandez-Lizarbe S, Montesinos J, Guerri C (2013). Ethanol induces TLR4/TLR2 association, triggering an inflammatory response in microglial cells. J Neurochem.

[81] Pascual-Lucas M, Fernandez-Lizarbe S, Montesinos J, Guerri C (2014). LPS or ethanol triggers clathrin- and rafts/caveolae-dependent endocytosis of TLR4 in cortical astrocytes. J Neurochem.

[82] Pascual M, Montesinos J, Montagud-Romero S (2017). TLR4 response mediates ethanol-induced neurodevelopment alterations in a model of fetal alcohol spectrum disorders. J Neuroinflammation.

[83] Shukla PK, Meena AS, Rao R, Rao RK (2018). Deletion of TLR-4 attenuates fetal alcohol exposure-induced gene expression and social interaction deficits. Alcohol.

[84] Goodfellow MJ, Shin YJ, Lindquist DH (2018). Mitigation of postnatal ethanol-induced neuroinflammation ameliorates trace fear memory deficits in juvenile rats. Behav Brain Res.

[85] Zhang K, Wang H, Xu M, Frank JA, Luo J (2018). Role of MCP-1 and CCR2 in ethanol-induced neuroinflammation and neurodegeneration in the developing brain. J Neuroinflammation.

[86] Cippitelli A, Domi E, Ubaldi M (2017). Protection against alcohol-induced neuronal and cognitive damage by the PPARgamma receptor agonist pioglitazone. Brain Behav Immun.

[87] Ullah I, Ullah N, Naseer MI, Lee HY, Kim MOK (2012). Neuroprotection with metformin and thymoquinone against ethanol-induced apoptotic neurodegeneration in prenatal rat cortical neurons. BMC Neurosci.

[88] Wang X, Zhang K, Yang F (2018). Minocycline protects developing brain against ethanol-induced damage. Neuropharmacology.

[89] Allis CD, Jenuwein T (2016). The molecular hallmarks of epigenetic control. Nat Rev Genet.

[90] Pinson MR, Tseng AM, Lehman TE (2023). Maternal circulating miRNAs contribute to negative pregnancy outcomes by altering placental transcriptome and fetal vascular dynamics. PLOS One.

[91] Kalisch-Smith JI, Moritz KM (2018). Detrimental effects of alcohol exposure around conception: Putative mechanisms. Biochem Cell Biol.

[92] Kaminen-Ahola N, Ahola A, Maga M (2010). Maternal ethanol consumption alters the epigenotype and the phenotype of offspring in a mouse model. PLOS Genet.

[93] McEwen BS (2017). Neurobiological and systemic effects of chronic stress. Chronic Stress.

[94] Schneider ML, Moore CF, Adkins MM (2011). The effects of prenatal alcohol exposure on behavior: Rodent and primate studies. Neuropsychol Rev.

[95] Wallén E, Auvinen P, Kaminen-Ahola N (2021). The effects of early prenatal alcohol exposure on epigenome and embryonic development. Genes.

[96] Masemola ML, van der Merwe L, Lombard Z, Viljoen D, Ramsay M (2015). Reduced DNA methylation at the PEG3 DMR and KvDMR1 loci in children exposed to alcohol in utero: A South African Fetal Alcohol Syndrome cohort study. Front Genet.

[97] Auvinen P, Vehviläinen J, Marjonen H (2022). Chromatin modifier developmental pluripotency associated factor 4 (DPPA4) is a candidate gene for alcohol-induced developmental disorders. BMC Med.

[98] Amiri S, Davie JR, Rastegar M (2020). Chronic ethanol exposure alters DNA methylation in neural stem cells: Role of mouse strain and sex. Mol Neurobiol.

[99] Lussier AA, Bodnar TS, Moksa M, Hirst M, Kobor MS, Weinberg J (2021). Prenatal adversity alters the epigenetic profile of the prefrontal cortex: Sexually dimorphic effects of prenatal alcohol exposure and food-related stress. Genes.

[100] Nordin M, Bergman D, Halje M, Engström W, Ward A (2014). Epigenetic regulation of the Igf2/H19 gene cluster. Cell Prolif.

[101] Haycock PC, Ramsay M (2009). Exposure of mouse embryos to ethanol during preimplantation development: Effect on DNA methylation in the h19 imprinting control region. Biol Reprod.

[102] Laufer BI, Mantha K, Kleiber ML, Diehl EJ, Addison SMF, Singh SM (2013). Long-lasting alterations to DNA methylation and ncRNAs could underlie the effects of fetal alcohol exposure in mice. Dis Model Mech.

[103] Portales-Casamar E, Lussier AA, Jones MJ (2016). DNA methylation signature of human fetal alcohol spectrum disorder. Epigenetics Chromatin.

[104] Marjonen H, Kahila H, Kaminen-Ahola N (2017). rs10732516 polymorphism at the IGF2/H19 locus associates with a genotype-specific trend in placental DNA methylation and head circumference of prenatally alcohol-exposed newborns. Hum Reprod Open.

[105] Carter RC, Chen J, Li Q (2018). Alcohol-related alterations in placental imprinted gene expression in humans mediate effects of prenatal alcohol exposure on postnatal growth. Alcohol Clin Exp Res.

[106] Ouko LA, Shantikumar K, Knezovich J, Haycock P, Schnugh DJ, Ramsay M (2009). Effect of alcohol consumption on CpG methylation in the differentially methylated regions of H19 and IG-DMR in male gametes: Implications for fetal alcohol spectrum disorders. Alcohol Clin Exp Res.

[107] Chang RC, Skiles WM, Chronister SS (2017). DNA methylation-independent growth restriction and altered developmental programming in a mouse model of preconception male alcohol exposure. Epigenetics.

[108] Chang RC, Wang H, Bedi Y, Golding MC (2019). Preconception paternal alcohol exposure exerts sex-specific effects on offspring growth and long-term metabolic programming. Epigenetics Chromatin.

[109] Bestry M, Symons M, Larcombe A (2022). Association of prenatal alcohol exposure with offspring DNA methylation in mammals: A systematic review of the evidence. Clin Epigenet.

[110] Subbanna S, Shivakumar M, Umapathy NS (2013). G9a-mediated histone methylation regulates ethanol-induced neurodegeneration in the neonatal mouse brain. Neurobiol Dis.

[111] Subbanna S, Nagre NN, Shivakumar M, Umapathy NS, Psychoyos D, Basavarajappa BS (2014). Ethanol induced acetylation of histone at G9a exon1 and G9a-mediated histone H3 dimethylation leads to neurodegeneration in neonatal mice. Neuroscience.

[112] Subbanna S, Basavarajappa BS (2014). Pre-administration of G9a/GLP inhibitor during synaptogenesis prevents postnatal ethanol-induced LTP deficits and neurobehavioral abnormalities in adult mice. Exp Neurol.

[113] Cantacorps L, Alfonso-Loeches S, Guerri C, Valverde O (2019). Long-term epigenetic changes in offspring mice exposed to alcohol during gestation and lactation. J Psychopharmacol.

[114] Shivakumar M, Subbanna S, Joshi V, Basavarajappa BS (2020). Postnatal ethanol exposure activates HDAC-mediated histone deacetylation, impairs synaptic plasticity gene expression and behavior in mice. Int J Neuropsychopharmacol.

[115] Li Y, Yuan F, Wu T (2019). Sulforaphane protects against ethanol-induced apoptosis in neural crest cells through restoring epithelial-mesenchymal transition by epigenetically modulating the expression of Snail1. Biochim Biophys Acta Mol Basis Dis.

[116] Chastain LG, Franklin T, Gangisetty O (2019). Early life alcohol exposure primes hypothalamic microglia to later-life hypersensitivity to immune stress: Possible epigenetic mechanism. Neuropsychopharmacology.

[117] Rachdaoui N, Li L, Willard B, Kasumov T, Previs S, Sarkar D (2017). Turnover of histones and histone variants in postnatal rat brain: Effects of alcohol exposure. Clin Epigenet.

[118] Miller MW (2003). Balance of cell proliferation and death among dynamic populations: A mathematical model. J Neurobiol.

[119] Chang RC, Thomas KN, Mehta NA, Veazey KJ, Parnell SE, Golding MC (2021). Programmed suppression of oxidative phosphorylation and mitochondrial function by gestational alcohol exposure correlate with widespread increases in H3K9me2 that do not suppress transcription. Epigenetics Chromatin.

[120] Veazey KJ, Parnell SE, Miranda RC, Golding MC (2015). Dose-dependent alcohol-induced alterations in chromatin structure persist beyond the window of exposure and correlate with fetal alcohol syndrome birth defects. Epigenetics Chromatin.

[121] Veazey KJ, Wang H, Bedi YS, Skiles WM, Chang RCA, Golding MC (2017). Disconnect between alcohol-induced alterations in chromatin structure and gene transcription in a mouse embryonic stem cell model of exposure. Alcohol.

[122] Khalid O, Kim JJ, Kim HS (2014). Gene expression signatures affected by alcohol-induced DNA methylomic deregulation in human embryonic stem cells. Stem Cell Res.

[123] Perkins A, Lehmann C, Lawrence RC, Kelly SJ (2013). Alcohol exposure during development: Impact on the epigenome. Int J Dev Neurosci.

[124] Nagre NN, Subbanna S, Shivakumar M, Psychoyos D, Basavarajappa BS (2015). CB1-receptor knockout neonatal mice are protected against ethanol-induced impairments of DNMT1, DNMT3A, and DNA methylation. J Neurochem.

[125] Liyanage VRB, Zachariah RM, Davie JR, Rastegar M (2015). Ethanol deregulates Mecp2/MeCP2 in differentiating neural stem cells via interplay between 5-methylcytosine and 5-hydroxymethylcytosine at the Mecp2 regulatory elements. Exp Neurol.

[126] Clarke S, Banfield K, Carmel R, Jacobsen DW (2001). S-Adenosylmethionine-dependent methyltransferases. Homocysteine in Health and Disease.

[127] Petry HG, Saini N, Smith SM, Mooney SM (2025). Alcohol exposure may increase prenatal choline needs through redirection of choline into lipid synthesis rather than methyl donation. Metabolites.

[128] Kable JA, Coles CD, Keen CL (2015). The impact of micronutrient supplementation in alcohol-exposed pregnancies on information processing skills in Ukrainian infants. Alcohol.

[129] Lussier AA, Bodnar TS, Mingay M (2018). Prenatal alcohol exposure: Profiling developmental DNA methylation patterns in central and peripheral tissues. Front Genet.

[130] Lussier AA, Morin AM, MacIsaac JL (2018). DNA methylation as a predictor of fetal alcohol spectrum disorder. Clin Epigenet.

[131] Krzyzewska IM, Lauffer P, Mul AN (2023). Expression quantitative trait methylation analysis identifies whole blood molecular footprint in fetal alcohol spectrum disorder (FASD). Int J Mol Sci.

[132] Loke YJ, Muggli E, Saffery R (2021). Sex- and tissue-specific effects of binge-level prenatal alcohol consumption on DNA methylation at birth. Epigenomics.

[133] Legault LM, Dupas T, Breton-Larrivée M (2024). Sex-specific DNA methylation and gene expression changes in mouse placentas after early preimplantation alcohol exposure. Environ Int.

[134] McBride N, Johnson S (2016). Fathers’ role in alcohol-exposed pregnancies: Systematic review of human studies. Am J Prev Med.

[135] Klonoff-Cohen H, Lam-Kruglick P, Gonzalez C (2003). Effects of maternal and paternal alcohol consumption on the success rates of in vitro fertilization and gamete intrafallopian transfer. Fertil Steril.

[136] Henriksen TB, Hjollund NH, Jensen TK (2004). Alcohol consumption at the time of conception and spontaneous abortion. Am J Epidemiol.

[137] Windham GC, Fenster L, Hopkins B, Swan SH (1995). The association of moderate maternal and paternal alcohol consumption with birthweight and gestational age. Epidemiology.

[138] Steinberger EK, Ferencz C, Loffredo CA (2002). Infants with single ventricle: A population-based epidemiological study. Teratology.

[139] Zuccolo L, DeRoo LA, Wills AK (2016). Pre-conception and prenatal alcohol exposure from mothers and fathers drinking and head circumference: Results from the Norwegian Mother-Child Study (MoBa). Sci Rep.

[140] Zhou Q, Song L, Chen J (2021). Association of preconception paternal alcohol consumption with increased fetal birth defect risk. JAMA Pediatr.

[141] May PA, Hasken JM, de Vries MM (2023). Maternal and paternal risk factors for fetal alcohol spectrum disorders: Alcohol and other drug use as proximal influences. Alcohol Clin Exp Res.

[142] Bakhireva LN, Wilsnack SC, Kristjanson A (2011). Paternal drinking, intimate relationship quality, and alcohol consumption in pregnant Ukrainian women. J Stud Alcohol Drugs.

[143] Anderson RA, Furby JE, Oswald C, Zaneveld LJ (1981). Teratological evaluation of mouse fetuses after paternal alcohol ingestion. Neurobehav Toxicol Teratol.

[144] Tanaka H, Suzuki N, Arima M (1982). Experimental studies on the influence of male alcoholism on fetal development. Brain Dev.

[145] Higgins SL, Bhadsavle SS, Gaytan MN, Thomas KN, Golding MC (2024). Chronic paternal alcohol exposures induce dose-dependent changes in offspring craniofacial shape and symmetry. Front Cell Dev Biol.

[146] Thomas KN, Srikanth N, Bhadsavle SS (2023). Preconception paternal ethanol exposures induce alcohol-related craniofacial growth deficiencies in fetal offspring. J Clin Invest.

[147] Chang RC, Thomas KN, Bedi YS, Golding MC (2019). Programmed increases in LXRα induced by paternal alcohol use enhance offspring metabolic adaptation to high-fat diet induced obesity. Mol Metab.

[148] Basel A, Bhadsavle SS, Scaturro KZ (2024). Parental alcohol exposures associate with lasting mitochondrial dysfunction and accelerated aging in a mouse model. Aging Dis.

[149] Di Micco R, Krizhanovsky V, Baker D, d’Adda di Fagagna F (2021). Cellular senescence in ageing: From mechanisms to therapeutic opportunities. Nat Rev Mol Cell Biol.

[150] Abel EL (1989). Paternal and maternal alcohol consumption: Effects on offspring in two strains of rats. Alcohol Clin Exp Res.

[151] Conner KE, Bottom RT, Huffman KJ (2020). The impact of paternal alcohol consumption on offspring brain and behavioral development. Alcohol Clin Exp Res.

[152] Beeler E, Nobile ZL, Homanics GE (2019). Paternal preconception every-other-day ethanol drinking alters behavior and ethanol consumption in offspring. Brain Sci.

[153] Liang F, Diao L, Jiang N (2015). Chronic exposure to ethanol in male mice may be associated with hearing loss in offspring. Asian J Androl.

[154] Bedi YS, Wang H, Thomas KN (2022). Alcohol induced increases in sperm histone H3 lysine 4 trimethylation correlate with increased placental CTCF occupancy and altered developmental programming. Sci Rep.

[155] Rompala GR, Mounier A, Wolfe CM, Lin Q, Lefterov I, Homanics GE (2018). Heavy chronic intermittent ethanol exposure alters small noncoding RNAs in mouse sperm and epididymosomes. Front Genet.

[156] Bedi Y, Chang RC, Gibbs R, Clement TM, Golding MC (2019). Alterations in sperm-inherited noncoding RNAs associate with late-term fetal growth restriction induced by preconception paternal alcohol use. Reprod Toxicol.

[157] Roach AN, Bhadsavle SS, Higgins SL (2024). Alterations in sperm RNAs persist after alcohol cessation and correlate with epididymal mitochondrial dysfunction. Andrology.

[158] Aye ILMH, Aiken CE, Charnock-Jones DS, Smith GCS (2022). Placental energy metabolism in health and disease-significance of development and implications for preeclampsia. Am J Obstet Gynecol.

[159] Carter RC, Wainwright H, Molteno CD (2016). Alcohol, methamphetamine, and marijuana exposure have distinct effects on the human placenta. Alcohol Clin Exp Res.

[160] Kwan STC, Presswood BH, Helfrich KK, Baulch JW, Mooney SM, Smith SM (2020). An interaction between fetal sex and placental weight and efficiency predicts intrauterine growth in response to maternal protein insufficiency and gestational exposure window in a mouse model of FASD. Biol Sex Differ.

[161] Thomas KN, Zimmel KN, Basel A (2022). Paternal alcohol exposures program intergenerational hormetic effects on offspring fetoplacental growth. Front Cell Dev Biol.

[162] Kwan STC, Ricketts DK, Presswood BH, Smith SM, Mooney SM (2021). Prenatal choline supplementation during mouse pregnancy has differential effects in alcohol-exposed fetal organs. Alcohol Clin Exp Res.

[163] Steane SE, Fielding AM, Kent NL (2021). Maternal choline supplementation in a rat model of periconceptional alcohol exposure: Impacts on the fetus and placenta. Alcohol Clin Exp Res.

[164] Apicella C, Ruano CSM, Méhats C, Miralles F, Vaiman D (2019). The role of epigenetics in placental development and the etiology of preeclampsia. Int J Mol Sci.

[165] Xu Y, Xiao R, Li Y (2005). Effect of ethanol on the development of visceral yolk sac. Hum Reprod.

[166] Gualdoni GS, Jacobo PV, Barril C, Ventureira MR, Cebral E (2021). Early abnormal placentation and evidence of vascular endothelial growth factor system dysregulation at the feto-maternal interface after periconceptional alcohol consumption. Front Physiol.

[167] Bolnick AD, Bolnick JM, Kohan-Ghadr HR (2018). Nifedipine prevents apoptosis of alcohol-exposed first-trimester trophoblast cells. Alcohol Clin Exp Res.

[168] Lui S, Jones RL, Robinson NJ, Greenwood SL, Aplin JD, Tower CL (2014). Detrimental effects of ethanol and its metabolite acetaldehyde, on first trimester human placental cell turnover and function. PLOS One.

[169] Tseng AM, Mahnke AH, Wells AB (2019). Maternal circulating miRNAs that predict infant FASD outcomes influence placental maturation. Life Sci Alliance.

[170] Gundogan F, Elwood G, Longato L (2008). Impaired placentation in fetal alcohol syndrome. Placenta.

[171] Fatayerji N, Engelmann GL, Myers T, Handa RJ (1996). In utero exposure to ethanol alters mRNA for insulin-like growth factors and insulin-like growth factor-binding proteins in placenta and lung of fetal rats. Alcohol Clin Exp Res.

[172] Tai M, Piskorski A, Kao JCW, Hess LA, de la Monte MSM, Gündoğan F (2017). Placental morphology in Fetal alcohol Spectrum Disorders. Alcohol Alcohol.

[173] Lo JO, Schabel MC, Roberts VHJ (2017). First trimester alcohol exposure alters placental perfusion and fetal oxygen availability affecting fetal growth and development in a non-human primate model. Am J Obstet Gynecol.

[174] Jakoubek V, Hampl V (2018). Alcohol and fetoplacental vasoconstrictor reactivity. Physiol Res.

[175] Gundogan F, Gilligan J, Qi W, Chen E, Naram R, de la Monte SM (2015). Dose effect of gestational ethanol exposure on placentation and fetal growth. Placenta.

[176] Ramadoss J, Jobe SO, Magness RR (2011). Alcohol and maternal uterine vascular adaptations during pregnancy-part I: Effects of chronic in vitro binge-like alcohol on uterine endothelial nitric oxide system and function. Alcohol Clin Exp Res.

[177] Saini N, Mooney SM, Smith SM (2023). Alcohol blunts pregnancy-mediated insulin resistance and reduces fetal brain glucose despite elevated fetal gluconeogenesis, and these changes associate with fetal weight outcomes. FASEB J.

[178] Saini N, Mooney SM, Smith SM (2025). Alcohol reprograms placental glucose and lipid metabolism, which correlate with reduced fetal brain but not body weight in a mouse model of prenatal alcohol exposure. J Nutr.

[179] Karl PI, Fisher SE (1994). Chronic ethanol exposure inhibits insulin and IGF-1 stimulated amino acid uptake in cultured human placental trophoblasts. Alcohol Clin Exp Res.

[180] Hutson JR, Stade B, Lehotay DC, Collier CP, Kapur BM (2012). Folic acid transport to the human fetus is decreased in pregnancies with chronic alcohol exposure. PLOS One.

[181] Falconer J (1990). The effect of maternal ethanol infusion on placental blood flow and fetal glucose metabolism in sheep. Alcohol Alcohol.

[182] Siler-Khodr TM, Yang Y, Grayson MH, Henderson GI, Lee M, Schenker S (2000). Effect of ethanol on thromboxane and prostacyclin production in the human placenta. Alcohol.

[183] Janeski JD, Naik VD, Carabulea AL, Jiang H, Ramadoss J (2024). In vivo administration of phosphatidic acid, a direct alcohol target rescues fetal growth restriction and maternal uterine artery dysfunction in rat FASD model. Nutrients.

[184] Haghighi Poodeh S, Salonurmi T, Nagy I (2012). Alcohol-induced premature permeability in mouse placenta-yolk sac barriers in vivo. Placenta.

[185] Holbrook BD, Davies S, Cano S (2019). The association between prenatal alcohol exposure and protein expression in human placenta. Birth Defects Res.

[186] Deyssenroth MA, Williams RP, Lesseur C (2024). Prenatal alcohol exposure is associated with changes in placental gene co-expression networks. Sci Rep.

[187] Chau K, Hennessy A, Makris A (2017). Placental growth factor and pre-eclampsia. J Hum Hypertens.

[188] Lecuyer M, Laquerrière A, Bekri S (2017). PLGF, a placental marker of fetal brain defects after in utero alcohol exposure. Acta Neuropathol Commun.

[189] Sautreuil C, Lecointre M, Dalmasso J (2023). Expression of placental CD146 is dysregulated by prenatal alcohol exposure and contributes in cortical vasculature development and positioning of vessel-associated oligodendrocytes. Front Cell Neurosci.

[190] Jégou S, El Ghazi F, de Lendeu PK (2012). Prenatal alcohol exposure affects vasculature development in the neonatal brain. Ann Neurol.

[191] Sautreuil C, Lecointre M, Derambure C (2023). Prenatal alcohol exposure impairs the placenta-cortex transcriptomic signature, leading to dysregulation of angiogenic pathways. Int J Mol Sci.

[192] Pinson MR, Bake S, Hurst DA, Samiya NT, Sohrabji F, Miranda RC (2023). Prenatal alcohol alters inflammatory signatures in enteric portal tissues following adult-onset cerebrovascular ischemic stroke. iScience.

[193] Williams RP, Lesseur C, Cheng H (2024). RNA-seq analysis reveals prenatal alcohol exposure is associated with placental inflammatory cells and gene expression. Gene.

[194] Masehi-Lano JJ, Deyssenroth M, Jacobson SW (2023). Alterations in placental inflammation-related gene expression partially mediate the effects of prenatal alcohol consumption on maternal iron homeostasis. Nutrients.

[195] Kwan STC, Kezer CA, Helfrich KK (2020). Maternal iron nutriture modulates placental development in a rat model of fetal alcohol spectrum disorder. Alcohol.

[196] Terasaki LS, Schwarz JM (2016). Effects of moderate prenatal alcohol exposure during early gestation in rats on inflammation across the maternal-fetal-immune interface and later-life immune function in the offspring. J Neuroimmune Pharmacol.

[197] Kim CJ, Romero R, Chaemsaithong P, Kim JS (2015). Chronic inflammation of the placenta: Definition, classification, pathogenesis, and clinical significance. Am J Obstet Gynecol.

[198] Goldstein JA, Gallagher K, Beck C, Kumar R, Gernand AD (2020). Maternal-fetal inflammation in the placenta and the Developmental Origins of Health and Disease. Front Immunol.

[199] Panzer JJ, Romero R, Greenberg JM (2023). Is there a placental microbiota? A critical review and re-analysis of published placental microbiota datasets. BMC Microbiol.

[200] Solmonson A, Faubert B, Gu W (2022). Compartmentalized metabolism supports midgestation mammalian development. Nature.

[201] Seitz HK, Moreira B, Neuman MG (2023). Pathogenesis of alcoholic fatty liver a narrative review. Life.

[202] Butte NF (2000). Carbohydrate and lipid metabolism in pregnancy: Normal compared with gestational diabetes mellitus. Am J Clin Nutr.

[203] Washburn SE, Sawant OB, Lunde ER, Wu G, Cudd TA (2013). Acute alcohol exposure, acidemia or glutamine administration impacts amino acid homeostasis in ovine maternal and fetal plasma. Amino Acids.

[204] Saini N, Virdee MS, Helfrich KK, Kwan STC, Mooney SM, Smith SM (2022). Untargeted metabolome analysis reveals reductions in maternal hepatic glucose and amino acid content that correlate with fetal organ weights in a mouse model of fetal alcohol spectrum disorders. Nutrients.

[205] Lopatynska-Mazurek M, Komsta L, Gibula-Tarlowska E, Kotlinska JH (2021). Aversive learning deficits and depressive-like behaviors are accompanied by an increase in oxidative stress in a rat model of fetal alcohol spectrum disorders: The protective effect of rapamycin. Int J Mol Sci.

[206] Steinberg GR, Hardie DG (2023). New insights into activation and function of the AMPK. Nat Rev Mol Cell Biol.

[207] Naik VD, Ramadoss J (2023). Untargeted and targeted blood lipidomic signature profile of gestational alcohol exposure. Nutrients.

[208] Välimäki M, Halmesmäki E, Keso L, Ylikorkala O, Ylikahri R (1990). Serum lipids and lipoproteins in alcoholic women during pregnancy. Metabolism.

[209] Padmanabhan R, Ibrahim A, Bener A (2002). Effect of maternal methionine pre-treatment on alcohol-induced exencephaly and axial skeletal dysmorphogenesis in mouse fetuses. Drug Alcohol Depend.

[210] Lunde-Young R, Davis-Anderson K, Naik V, Nemec M, Wu G, Ramadoss J (2018). Regional dysregulation of taurine and related amino acids in the fetal rat brain following gestational alcohol exposure. Alcohol.

[211] Sawant OB, Wu G, Washburn SE (2015). Maternal L-glutamine supplementation prevents prenatal alcohol exposure-induced fetal growth restriction in an ovine model. Amino Acids.

[212] Palis J (2024). Erythropoiesis in the mammalian embryo. Exp Hematol.

[213] Huebner SM, Blohowiak SE, Kling PJ, Smith SM (2016). Prenatal alcohol exposure alters fetal iron distribution and elevates hepatic hepcidin in a rat model of fetal alcohol spectrum disorders. J Nutr.

[214] Carter RC, Jacobson JL, Molteno CD (2012). Effects of heavy prenatal alcohol exposure and iron deficiency anemia on child growth and body composition through age 9 years. Alcohol Clin Exp Res.

[215] Georgieff MK (2023). The importance of iron deficiency in pregnancy on fetal, neonatal, and infant neurodevelopmental outcomes. Int J Gynaecol Obstet.

[216] Huebner SM, Helfrich KK, Saini N (2018). Dietary iron fortification normalizes fetal hematology, hepcidin, and iron distribution in a rat model of prenatal alcohol exposure. Alcohol Clin Exp Res.

[217] Helfrich KK, Saini N, Kwan STC, Rivera OC, Mooney SM, Smith SM (2023). Fetal anemia and elevated hepcidin in a mouse model of fetal alcohol spectrum disorder. Pediatr Res.

[218] Carter RC, Jacobson SW, Molteno CD, Jacobson JL (2007). Fetal alcohol exposure, iron-deficiency anemia, and infant growth. Pediatrics.

[219] Carter RC, Senekal M, Duggan CP (2022). Gestational weight gain and dietary energy, iron, and choline intake predict severity of fetal alcohol growth restriction in a prospective birth cohort. Am J Clin Nutr.

[220] Carter RC, Georgieff MK, Ennis KM (2021). Prenatal alcohol-related alterations in maternal, placental, neonatal, and infant iron homeostasis. Am J Clin Nutr.

[221] Roberts H, Woodman AG, Baines KJ, Jeyarajah MJ, Bourque SL, Renaud SJ (2021). Maternal iron deficiency alters trophoblast differentiation and placental development in rat pregnancy. Endocrinology.

[222] Rufer ES, Tran TD, Attridge MM, Andrzejewski ME, Flentke GR, Smith SM (2012). Adequacy of maternal iron status protects against behavioral, neuroanatomical, and growth deficits in fetal alcohol spectrum disorders. PLOS One.

[223] Huebner SM, Tran TD, Rufer ES, Crump PM, Smith SM (2015). Maternal iron deficiency worsens the associative learning deficits and hippocampal and cerebellar losses in a rat model of fetal alcohol spectrum disorders. Alcohol Clin Exp Res.

[224] Carter RC, Jacobson JL, Burden MJ (2010). Iron deficiency anemia and cognitive function in infancy. Pediatrics.

[225] Cantor AG, Holmes R, Bougatsos C, Atchison C, DeLoughery T, Chou R (2024). Screening and supplementation for iron deficiency and iron deficiency anemia during pregnancy: Updated evidence report and systematic review for the US Preventive Services Task Force. JAMA.

[226] Iriarte-Gahete M, Tarancon-Diez L, Garrido-Rodríguez V, Leal M, Pacheco YM (2024). Absolute and functional iron deficiency: Biomarkers, impact on immune system, and therapy. Blood Rev.

[227] Miller MW, Roskams AJ, Connor JR (1995). Iron regulation in the developing rat brain: Effect of in utero ethanol exposure. J Neurochem.

[228] De la Fuente-Ortega E, Plaza-Briceño W, Vargas-Robert S, Haeger P (2019). Prenatal ethanol exposure misregulates genes involved in iron homeostasis promoting a maladaptation of iron dependent hippocampal synaptic transmission and plasticity. Front Pharmacol.

[229] Sozo F, Dick AM, Bensley JG (2013). Alcohol exposure during late ovine gestation alters fetal liver iron homeostasis without apparent dysmorphology. Am J Physiol Regul Integr Comp Physiol.

[230] Hasken JM, de Vries MM, Marais AS, May PA (2024). Contribution of ferritin and zinc to adverse infant outcomes among pregnancies with prenatal alcohol exposure in South Africa. Reprod Toxicol.

[231] Conde Díez S, de Las Cuevas Allende R, Conde García E (2024). Anemia of inflammation and iron metabolism in chronic diseases. Rev Clin Esp.

[232] Saini N, Helfrich KK, Kwan STC (2019). Alcohol’s dysregulation of maternal-fetal IL-6 and p-STAT3 is a function of maternal iron status. Alcohol Clin Exp Res.

[233] Gambling L, Czopek A, Andersen HS (2009). Fetal iron status regulates maternal iron metabolism during pregnancy in the rat. Am J Physiol Regul Integr Comp Physiol.

[234] Helfrich KK, Saini N, Kwan STC, Rivera OC, Hodges R, Smith SM (2022). Gestational iron supplementation improves fetal outcomes in a rat model of prenatal alcohol exposure. Nutrients.

[235] Weiss G, Ganz T, Goodnough LT (2019). Anemia of inflammation. Blood.

[236] Ganz T, Locatelli F, Arici M, Akizawa T, Reusch M (2023). Iron parameters in patients treated with Roxadustat for anemia of chronic kidney disease. J Clin Med.

[237] Kampen KR, Sulima SO, Vereecke S, De Keersmaecker K (2020). Hallmarks of ribosomopathies. Nucleic Acids Res.

[238] Flentke GR, Wilkie TE, Baulch J, Huang Y, Smith SM (2024). Alcohol exposure suppresses ribosome biogenesis and causes nucleolar stress in cranial neural crest cells. PLOS One.

[239] Huang Y, Flentke GR, Rivera OC, Saini N, Mooney SM, Smith SM (2024). Alcohol exposure induces nucleolar stress and apoptosis in mouse neural stem cells and late-term fetal brain. Cells.

[240] Jin S, Cao Q, Yang F (2021). Brain ethanol metabolism by astrocytic ALDH2 drives the behavioural effects of ethanol intoxication. Nat Metab.

[241] Hodo TW, de Aquino MTP, Shimamoto A, Shanker A (2020). Critical neurotransmitters in the neuroimmune network. Front Immunol.

[242] Mittal R, Debs LH, Patel AP (2017). Neurotransmitters: The critical modulators regulating gut-brain axis. J Cell Physiol.

